# Convergent Active Site Evolution in Platinum Single Atom Catalysts for Acetylene Hydrochlorination and Implications for Toxicity Minimization

**DOI:** 10.1021/acscatal.4c03533

**Published:** 2024-08-29

**Authors:** V. Giulimondi, M. Vanni, S. Damir, T. Zou, S. Mitchell, F. Krumeich, A. Ruiz-Ferrando, N. López, J.J. Gata-Cuesta, G. Guillén-Gosálbez, J.J. Smit, P. Johnston, J. Pérez-Ramírez

**Affiliations:** † Department of Chemistry and Applied Biosciences, ETH Zürich, Vladimir-Prelog-Weg 1, Zürich 8093, Switzerland; ‡ Institute of Chemical Research of Catalonia (ICIQ-CERCA), Tarragona 43007, Spain; § University of Rovira i Virgili, Av. Catalunya 35, Tarragona 43002, Spain; ∥ Johnson Matthey, Catalyst Technologies, Eastbourne Terrace 10, London W2 6LG, U.K.; ⊥ Johnson Matthey, Catalyst Technologies, Belasis Avenue 1, Billingham TS23 1LB, U.K.

**Keywords:** single atom catalysis, acetylene hydrochlorination, platinum, active site dynamics, toxicity, kinetic modeling

## Abstract

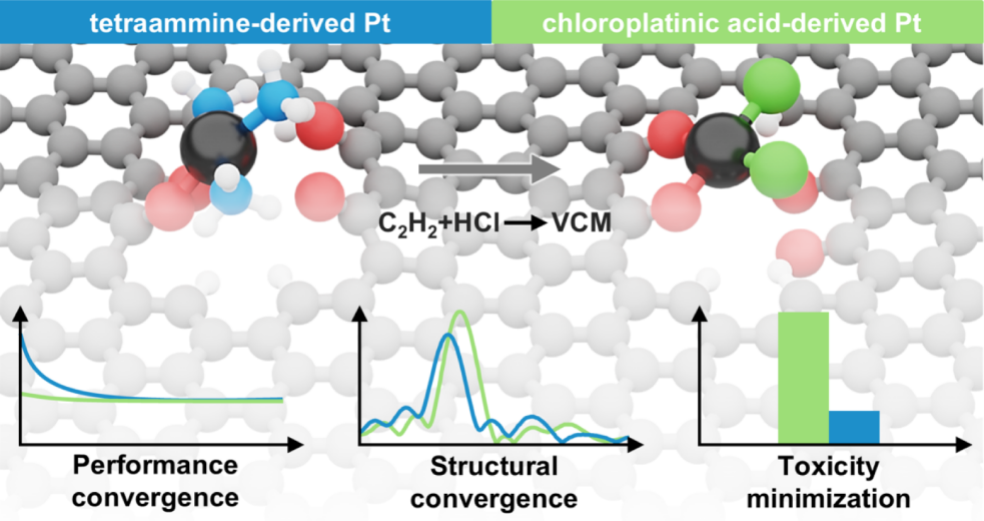

Platinum single atoms anchored onto activated carbon enable highly stable Hg-free synthesis of vinyl chloride (VCM) via acetylene hydrochlorination. Compared to gold-based alternatives, platinum catalysts are in initial phases of development. Most synthetic approaches rely on chloroplatinic acid, presenting opportunities to explore other precursors and their impact on catalyst structure, reactivity, and toxicity aspects. Here, we synthesize platinum single atom catalysts (Pt SACs, 0.2–0.8 wt % Pt) employing diverse Pt^2+^ and Pt^4+^ complexes with ammine, hydroxyl, nitrate, and chloride ligands, following a scalable impregnation protocol on activated carbon extrudates. X-ray absorption spectroscopy (XAS) reveals that Pt^4+^ species reduce to Pt^2+^ upon deposition onto the support. Despite similar oxidation states, the initial activity is precursor dependent, with tetraammine-derived Pt SACs displaying 2-fold higher VCM yield than chlorinated counterparts, linked to superior hydrogen chloride binding abilities by density functional theory (DFT) simulations. Their activity gradually converges due to dynamic active site restructuring, delivering remarkable precursor-independent stability over 150 h. *Operando* XAS and DFT studies uncover reaction-induced ligand exchange, generating common active and stable Pt–Cl*_x_* (*x* = 2–3) species. Convergent active site evolution enables flexibility in metal precursor selection and thus toxicity minimization through multiparameter assessment. This study advances safe-by-design catalysts for VCM synthesis, highlighting the importance of toxicity analyses in early-stage catalyst development programs.

## Introduction

The synthesis of vinyl chloride monomer (VCM) via acetylene hydrochlorination, accounting for 30% of global poly(vinyl chloride) production, is a long-established industrial process that relies on highly toxic and volatile catalysts consisting of HgCl_2_ supported on activated carbon.^1,2^ Decades of research into more sustainable alternatives have identified metals such as Au, Pd, Cu, and Ru, mainly in chloride form, as promising candidates, demonstrating a strong dependence of catalytic performance on the metal nanostructure.^3−8^ In Au-based catalysts, which exhibit the highest initial VCM productivity among metal chlorides, single Au–Cl cations are the active species,^8^ although they tend to deactivate through sintering into inactive nanoparticles. To address this drawback, synthesis strategies integrating soft-donor ligands (thiosulfate, thiocyanate, thiourea, and cyanides) could improve the stability of Au cations,^9−11^ leading to the commercialization of an activated carbon-supported Au catalyst derived from a thiosulfate precursor ((NH_4_)_2_Au(S_2_O_3_)_3_).^11^ Notably, ligand-modification strategies have successfully resulted in improved metal dispersion and resistance to reduction and sintering also for carbon-supported Pd, Cu, and Ru catalysts.^12−14^

Initially overlooked owing to the lower activity of the metal chloride, Pt nanostructured as chlorinated single atoms on activated carbon has enabled exceptionally stable performance, showing promise for further optimization.^15,16^ In contrast to Au-based systems, developed over decades of research, the design and implementation of Pt catalysts are in early stages and synthesis-structure-performance relations are still not well understood. Pt catalysts may find application in acetylene hydrochlorination technologies where the use of Au catalysts is either not advantageous or favored. Recent research has revealed the bifunctional synergy between carbon in binding acetylene and metal atoms, as Pt–Cl*_x_* (*x* = 2–3) species, in activating hydrogen chloride.^17^ However, chloroplatinic acid (CPA), a hazardous and highly sensitizing compound that can be irritating and damaging to the skin and respiratory system, has been almost exclusively used as the metal precursor for synthesizing carbon-supported Pt single atom catalysts (SACs).^6,15,18−23^ This leaves substantial opportunities for exploring other metal complexes, including halide-free ones, their impact on catalyst structure, performance, and other sustainability-related aspects. In this context, in-depth knowledge of (i) the structural evolution of the metal precursor upon deposition onto carbon, (ii) the reactivity of different metal–ligand architectures, and (iii) their dynamic behavior during reaction is central to advancing Pt SAC design and bring this technology closer to implementation.

Herein, we synthesize eight customized Pt SACs (0.2–0.8 wt % Pt) spanning from a variety of Pt^2+^ and Pt^4+^ precursor complexes featuring ammine, hydroxyl, nitrate, and chloride ligands. The employed impregnation protocol is standardized, scalable, and conducted on carbon extrudates, flavoring the degree of reality of our findings for the future application of these catalysts. Microscopy and spectroscopy analyses are conducted to confirm the atomic metal dispersion. X-ray absorption spectroscopy (XAS) enables us to monitor the evolution of the metal precursors’ oxidation and coordination environments upon deposition onto the support, all stabilizing as single atoms – featuring different ligands – in a state close to Pt^2+^. Catalytic tests are performed to explore initial activity trends across different metal–ligand architectures, which are linked to their hydrogen-chloride binding abilities by density functional theory (DFT) simulations. Pt SACs derived from tetraammine precursors show 2-fold higher initial activity than the CPA-derived reference. Dynamic metal restructuring leads to gradual convergence to the same VCM productivity, resulting in remarkable precursor-independent stability over 150 h. *Operando* XAS and DFT simulations are employed to gain detailed understanding of a reaction-induced ligand-exchange process resulting in common active and stable Pt–Cl*_x_* (*x* = 2–3) species. The convergent active site evolution enables flexibility in Pt precursor selection without compromising performance over extended time on stream, which can also bring advantages from a sustainability standpoint. We develop a multicriteria assessment to rank Pt precursors based on their toxicity, highlighting the importance of considering this factor in catalyst development programs.

## Methods

### Catalyst Preparation

The Pt SACs (nominal metal content of 0.2 or 0.8 wt %) were synthesized via incipient wetness impregnation, employing custom-synthesized metal precursors (H_2_PtCl_6_, K_2_PtCl_4_, Na_2_Pt(OH)_6_, Pt(NO_3_)_4_, [(NH_3_)_4_Pt]Cl_2_, [(NH_3_)_4_Pt]citrate, [(NH_3_)_4_Pt](HCO_3_)_2_, and [(NH_3_)_4_Pt]SO_4_) and water as solvent. The obtained solutions were added dropwise onto the commercial activated carbon (AC, Norit ROX 0.8) support with agitation and mixing. The obtained catalysts were dried at 383 K in air for 16 h. Further details of the catalyst synthesis are provided in the Supporting Information.

### Catalyst Characterization

The composition of the catalysts was evaluated by elemental composition analysis and inductively coupled plasma (ICP) optical emission spectrometry. The porous and structural properties of the AC extrudates were assessed by nitrogen sorption at 77 K and mercury porosimetry, together with microcomputed tomography and scanning electron microscopy with backscattered electrons (BSE-SEM), respectively. The metal dispersion was assessed through X-ray diffraction (XRD) and high-angle annular dark-field scanning transmission electron microscopy (HAADF-STEM). The chemical state of the metal atoms and the carbon supports were evaluated by X-ray photoelectron spectroscopy (XPS). The metal oxidation state and coordination environment under reactive environments, were monitored by operando X-ray absorption spectroscopy (XAS), respectively, by X-ray absorption near edge spectroscopy (XANES) and extended X-ray absorption fine structure (EXAFS). Coke deposits on the catalysts after use in acetylene hydrochlorination were quantified by thermogravimetric analysis (TGA). All characterization techniques and procedures are detailed in the Supporting Information.

### Catalyst Evaluation

The hydrochlorination of acetylene was evaluated at atmospheric pressure in a continuous-flow fixed-bed reactor setup, as described elsewhere.^15^ In a typical test, the catalyst (*W*_cat_ = 0.25 g) was loaded in the quartz reactor and heated in a He flow to the desired bed temperature (*T*_bed_ = 433–473 K). After stabilization for at least 15 min, the reaction mixture (40 vol % C_2_H_2_, 44 vol % HCl, and 16 vol % Ar) was fed at a total volumetric flow of *F*_T_ = 7.5–15 cm^3^ min^–1^. A detailed description of catalyst evaluation, product analysis, and kinetic studies and model derivation is provided in the Supporting Information.

### Computational Methods

To gain insights into the interaction of acetylene with distinct metal sites, DFT calculations were performed using the Vienna Ab initio Simulation Package with projector augmented wave core potentials and the PBE-D3 functional,^24 −27^ as detailed in the Supporting Information. In brief, five oxidic defects (i) tetraketone (keto_4_), (ii) monothiophene (S), and (ii) triketone-thiophene (keto_3_-S) coordination sites. The AC-supported Pt SACs were modeled by placing PtCl_2_, Pt(NH_3_)_2_, Pt(OH)_2_, and Pt(NO_3_)_2_ moieties at the center of the distinct AC coordination sites.

### Toxicity Evaluation

Information on the toxicity of the platinum precursors was gathered following the Classification, Labeling, and Packaging (CLP) regulation from the European Commission, overseen by the European Chemicals Agency (ECHA).^28^ This regulation classifies the hazards of a substance by assigning a certain hazard class and associated category code, with each category code qualitatively representing the hazard severity within the respective class. In this study, a total of 11 health and environmental hazard classes were considered. For each hazard class associated with a given platinum precursor, its category codes were sorted in a set from least to most severe according to their qualitative description in the CLP regulation. These category codes were mapped to a numerical value ranging from one to N, where N corresponds to the highest qualitative toxicity level within the hazard class. Hence, an overall toxicity score was assigned for a given metal precursor by aggregation of all hazard scores through normalization and application of weighting factors. Further details pertaining to evaluation of the toxicity, as well as reactivity,^29^ of the platinum precursors are provided in the Supporting Information.

## Results and Discussion

### Synthesis and Characterization of Platinum Single Atom Catalysts

**Figure 1:**
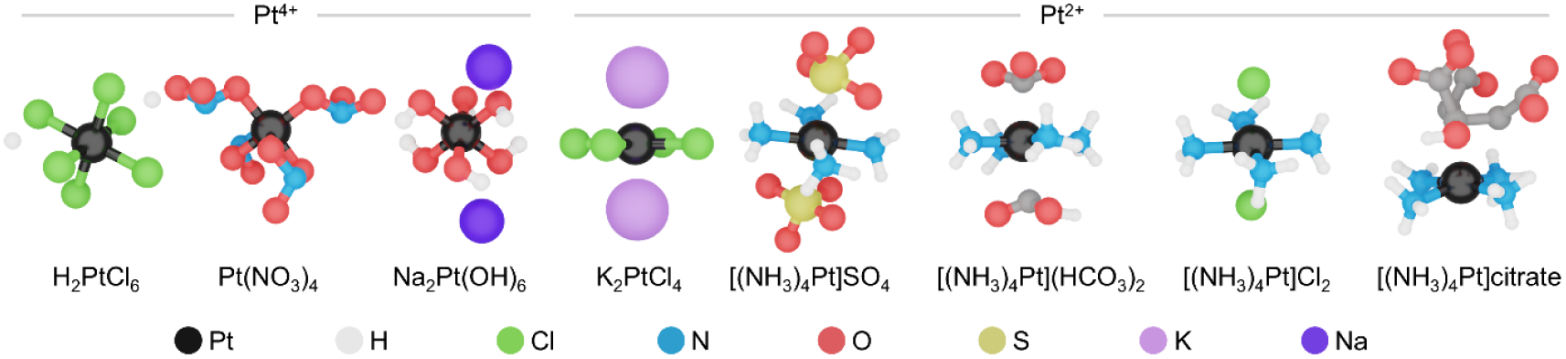
Structure of the metal precursors employed to synthesize Pt SACs for acetylene hydrochlorination.

**Figure 2:**
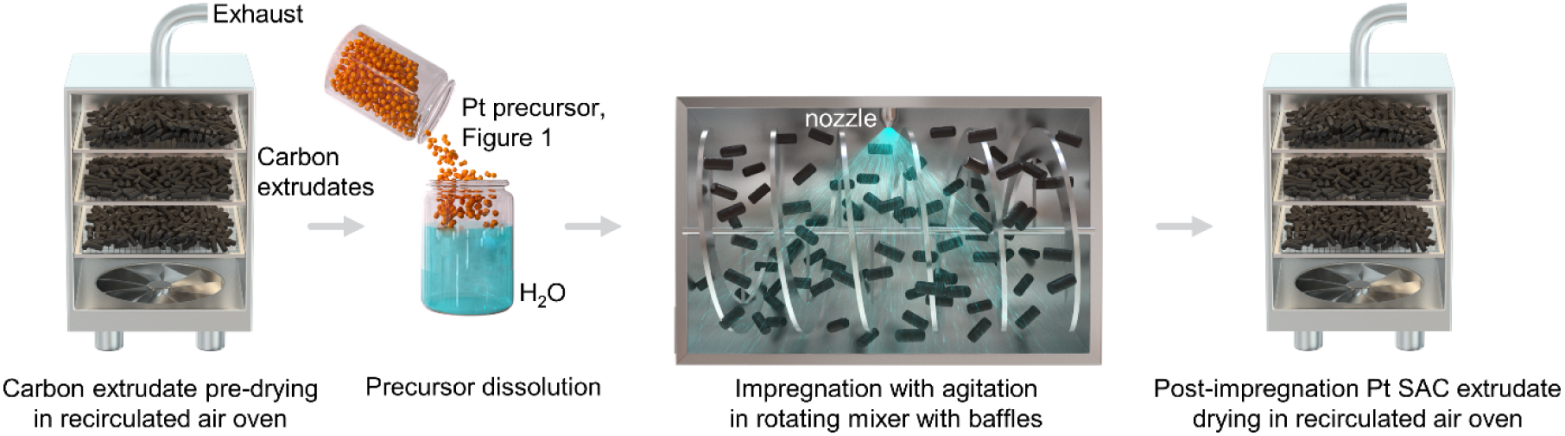
Schematic representations of the incipient wetness impregnation, approach to synthesize carbon-supported Pt SACs.

**Figure 3:**
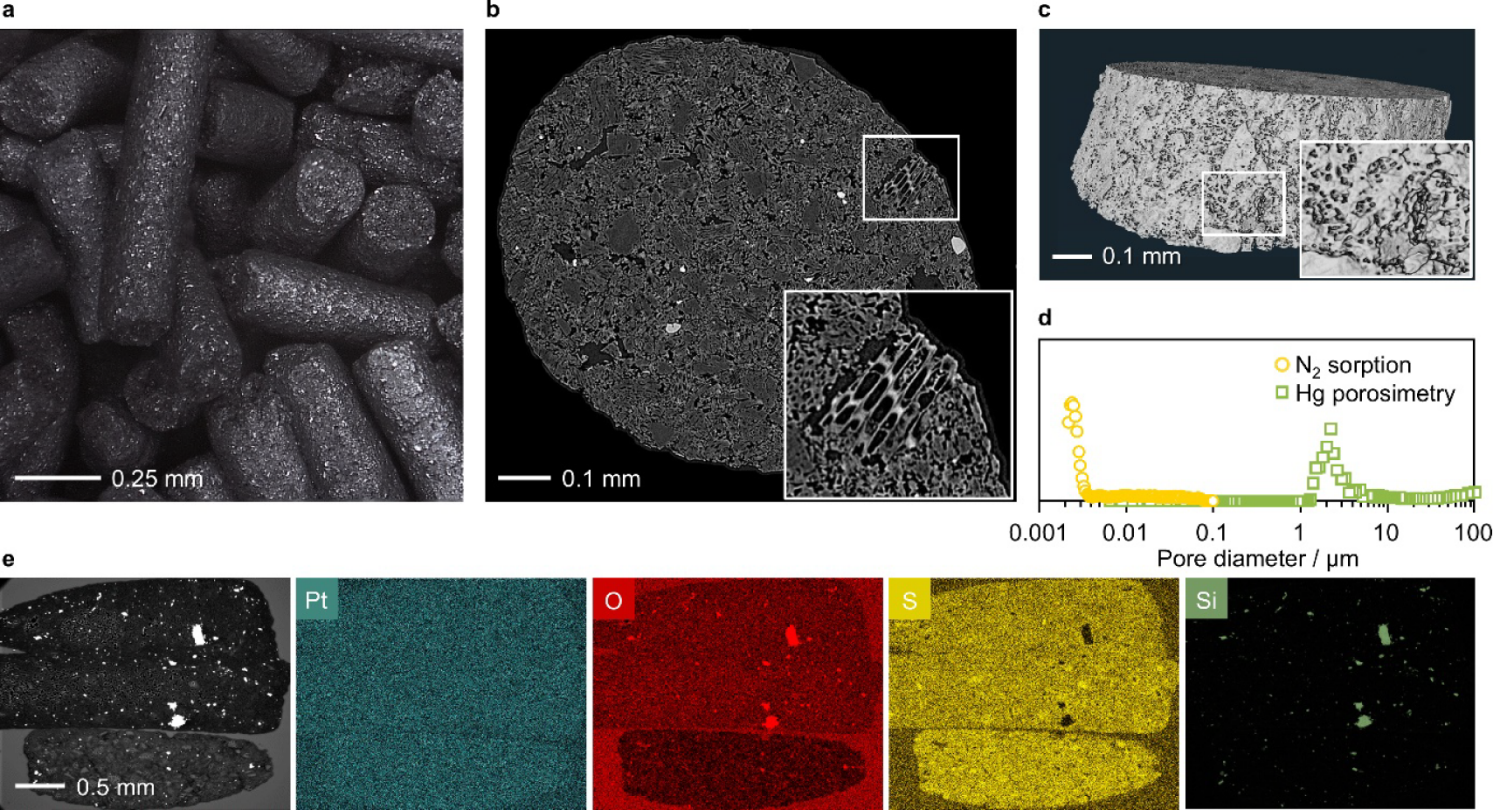
(a) Optical microscopy image, together with (b) virtual slice of a transversal cross-section, (c) 3D reconstruction of the macropore network obtained by microcomputed tomography, and (d) pore size distribution as determined by porosity analyses. (e) BSE-SEM image and EDX maps of most abundant elements in 0.2-[(NH_3_)_4_Pt]SO_4_.

To explore the effect of the oxidation and coordination states of the metal precursor, a variety of platinum complexes are selected for the synthesis of Pt SACs (Figure 1). These comprise a Pt^4+^ series including the literature-reported H_2_PtCl_6_ together with chloride-free Pt(NO_3_)_4_ and Na_2_Pt(OH)_6_; and a Pt^2+^ series featuring chloride ligands, K_2_PtCl_4_, and ammine ligands with diverse counterions, [(NH_3_)_4_Pt]SO_4_, [(NH_3_)_4_Pt](HCO_3_)_2_, [(NH_3_)_4_Pt]Cl_2_, [(NH_3_)_4_Pt]citrate. Of these, [(NH_3_)_4_Pt]SO_4_ and [(NH_3_)_4_Pt]citrate are custom-made for this study. All metal compounds were selected for their solubility in water, avoiding the need for acidic or organic solvents. We devise a standardized and scalable incipient wetness impregnation method, applied on carbon extrudates, i.e., support in suitable form for potential use in large-scale fixed-bed reactors. It involves the following steps (Figure 2): (i) predrying of the carbon extrudates in a recirculated air oven, (ii) their impregnation with the Pt precursor under agitation and mixing, and (iii) drying of the metal-containing extrudates. Catalysts with a nominal metal content of 0.2 or 0.8 wt %, confirmed by inductively coupled plasma optical emission spectrometry (ICP-OES, Table S1), are prepared and denoted as *X*–*M* (metal content *X* = 0.2 or 0.8; metal precursor, *M*). The employed support is an extruded commercial activated carbon (AC, Figure 3a), derived from steam activation of wood charcoal. The resulting AC extrudates exhibit a heterogeneous structure with amorphous and cellulosic regions together with a well-developed macropore network, as respectively visualized by microcomputed tomography analysis (micro-CT, Figure 3b,c). Alongside macropores of 1–5 μm, the AC extrudates integrate mesopores of 2–4 nm and micropores, as shown by Hg porosimetry and N_2_ sorption (Figures 3d and S1, Table S2). Furthermore, diverse impurities are present as detected by scanning electron microscopy with backscattered electrons (BSE-SEM) with energy-dispersive X-ray (EDX) analysis (Figures 3e and S2), such as SiO_2_ particles. Despite their heterogeneous structure, uniform platinum distribution across the AC extrudates is obtained with the developed impregnation synthesis approach (Figures 3e and S3).

**Figure 4:**
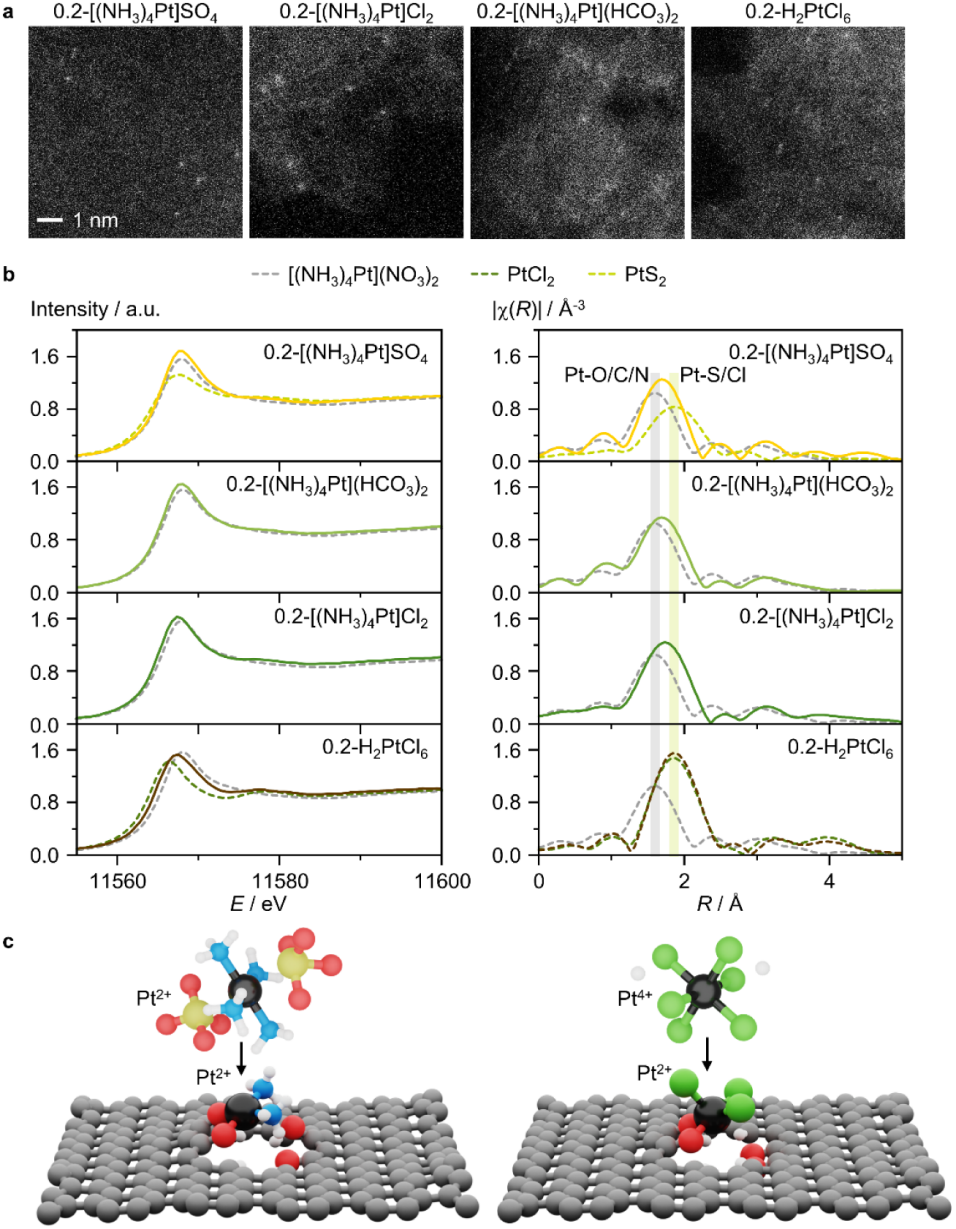
(a) HAADF-STEM images and (b) Pt *L*_3_ edge XANES (left) and EXAFS (right) of selected Pt SACs, together with spectra of reference compounds in dotted lines. Scale bar is the same in all images. (c) Schematic representation of the changes in the structure and metal oxidation state that different metal precursors undergo during deposition on carbon, as indicated by XANES and EXAFS analysis.

Atomic metal dispersion is corroborated by the absence of Pt reflections in X-ray diffraction (XRD, Figure S4) and the visualization of isolated atoms by high-angle annular dark-field scanning transmission electron microscopy (HAADF-STEM, Figures 4a, S5, and S6), for both the 0.2 and 0.8 wt % catalyst series. AC presents O-, N- and S-functionalities, as evidenced by elemental analysis (Table S1), that constitute potential anchoring sites for the metal atoms.^30,31^ X-ray photoelectron spectroscopy (XPS) indicates O-functionalities as the most abundant species on the catalyst surface (Table S3). Specifically, XPS analysis identifies the presence of oxidized Pt species likely anchored to keto (C=O) and thiophene (S) functionalities, together with inorganic chloride species probably deriving from metal salt impurities (Figure S7; Tables S4–S7).^32^ The stabilization of the metal species as single atoms is further confirmed by extended X-ray absorption fine structure (EXAFS) results, showing no metal–metal contributions (Figure 4b, Table S8). Furthermore, analysis of the first coordination shell of the Pt species by EXAFS enables quantitative assessment of scattering paths with light elements such as O, C, and N as well as with heavier elements such as Cl and S. Derived from the typically employed CPA precursor, 0.2-H_2_PtCl_6_ presents not only a Pt–Cl/S contribution (coordination number, CN = 3.9), mostly attributable to chloride ligands, but also a Pt–O/C/N one (CN = 0.4), consistent with metal anchoring on the support likely through O-functionalities. Chloride-free tetraammine-derived 0.2-[(NH_3_)_4_Pt]SO_4_, 0.2-[(NH_3_)_4_Pt](HCO_3_)_2_, and 0.2-[(NH_3_)_4_Pt]Cl_2_, present Pt–O/C/N contributions (CN = 3.8, 3.7, and 3.5, respectively) originating from both Pt-NH_3_ bonds as well as Pt anchoring on the support over O-functionalities, and also minor Pt–Cl/S contributions (CN = 0.7, 0.6, and 1.3, respectively) suggesting some Pt anchoring over S-functionalities or potential interaction with chloride counterions in the case of [(NH_3_)_4_Pt]Cl_2_. Notably, partial ligand loss is noted following the deposition of the metal precursors on the carbon. 0.2-H_2_PtCl_6_ shows ca. three chloride ligands as opposed to the six ligands present in the CPA precursor, while the tetraammine-derived Pt SACs present ca. three ammonia ligands. This phenomenon is assigned to the reducing nature of the carbon surface, which can displace ligands from the metal.^33^

Beyond the coordination environment, analysis of the X-ray absorption near edge structure (XANES) provides information on the electronic structure of the Pt species (Figure 4b). 0.2-[(NH_3_)_4_Pt]SO_4_, 0.2-[(NH_3_)_4_Pt](HCO_3_)_2_, and 0.2-[(NH_3_)_4_Pt]Cl_2_ present spectral features similar to the [(NH_3_)_4_Pt](NO_3_)_2_ reference compound, indicating that the Pt^2+^ oxidation state of the respective metal precursor is preserved upon impregnation. In contrast, the XANES of 0.2-H_2_PtCl_6_ resembles that of the PtCl_2_ reference compound (Figure 4c), indicating a reduction of the Pt^4+^ species to Pt^2+^ upon deposition of the H_2_PtCl_6_ precursor on the AC extrudates. This reflects the chloride-ligand loss (*vide supra*) observed in EXAFS analysis upon deposition of the high oxidation state CPA precursor. The stabilization of Pt^2+^ single atoms, regardless of whether the employed metal precursor features Pt^2+^ or Pt^4+^ species, is attributable to the presence of carbon surface functionalities that can be oxidized by Pt^4+^ species.^15,17,33^ This holds significance, as any differences in catalytic activity among samples are likely attributable to metal–ligand coordination rather than distinct metal oxidation states.

### Catalyst Performance

**Figure 5:**
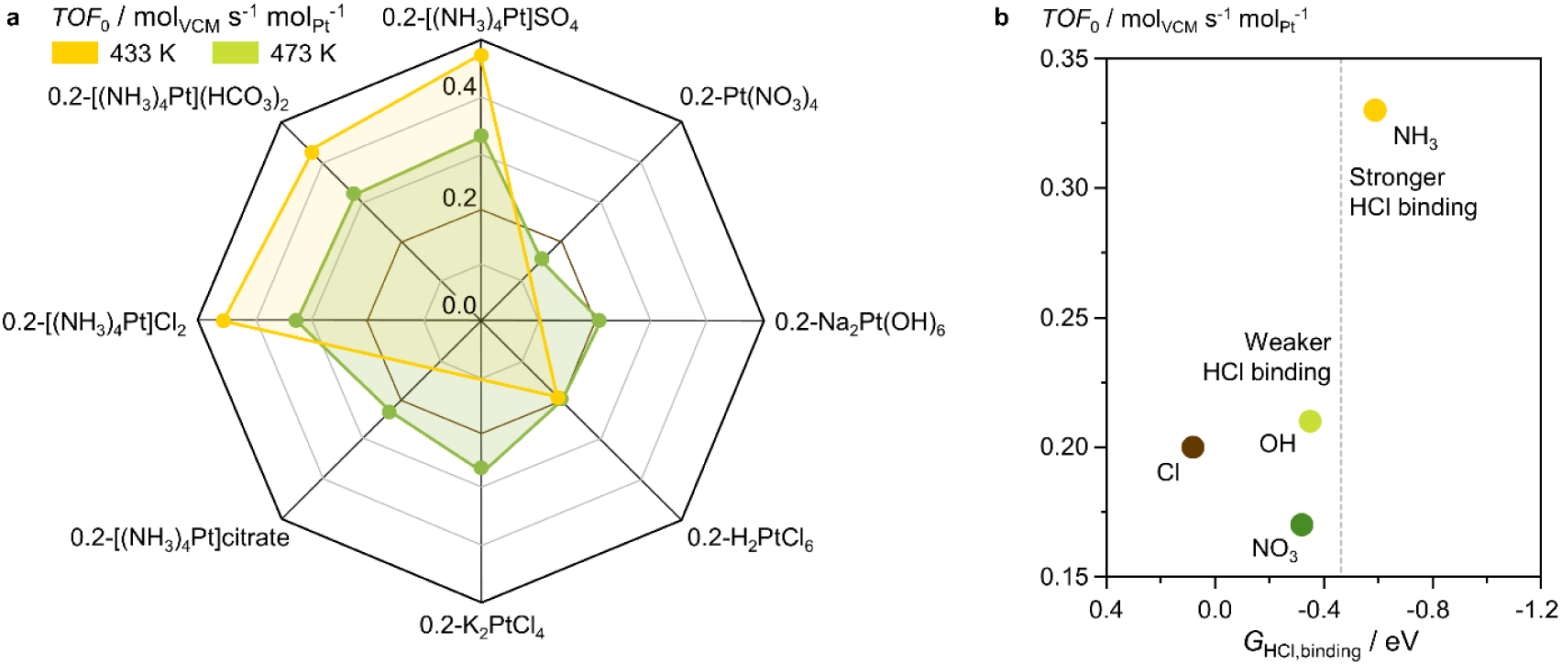
(a) Initial turnover frequency, *TOF*_0_, of the Pt SACs, where the brown line marks the level of activity of benchmark literature-reported Pt SAC derived from H_2_PtCl_6_ on the same commercial carbon, and (b) as a function of the HCl-binding Gibbs energy, *G*_HCl,binding_, of Pt*L*_2_ sites (*L* = NH_3_, NO_3_^–^, OH^–^, Cl^–^). Reaction conditions: *GHSV*(C_2_H_2_) = 650 h^–1^, C_2_H_2_:HCl:Ar = 40:44:16, *T* = 433 or 473 K, *P* = 1 bar.

The impact of the metal precursor on the performance of Pt SACs is evaluated in acetylene hydrochlorination to VCM (Figures 1 and 5a, Table S9). Considering cost minimization for catalyst manufacture, we focus on the 0.2 wt % Pt SAC series. Their initial turnover frequency (*TOF*_0_) is evaluated after 1 h on stream, at gas hourly space velocities for acetylene (*GHSV*(C_2_H_2_)) of 650 h^–1^ and temperatures of 433 and 473 K, as the operating temperature of the reaction typically lies in this range.^16^ Despite all exhibiting a state close to Pt^2+^, (*vide supra*, Figure 4c), the Pt SACs derived from the eight diverse Pt precursors show distinct initial activity (Figure 5a). This could be explained by different adaptive coordination of the Pt^2+^ species deriving from the precursor’s metal–ligand architecture and its binding to the carbon surface. While the carbon support is often considered as a ligand to the metal site, one should be mindful of the different structural and electronic properties that it exhibits compared with ions or molecules bearing functional groups, which are typically studied in coordination chemistry.^34^ Therefore, geometries of Pt single atoms on carbons may deviate from those that are well established in transition metal complexes. However, current limitations in characterization and computational techniques render the identification of such geometries highly uncertain.^35^ As a result, the initial activity trends observed in this study are discussed and tentatively related to the metal precursor structure under the assumption that the metal–ligand structure is partly preserved upon deposition on the carbon support and under reaction temperature (where some ligand loss may occur).

At 473 K, 0.2-Pt(NO_3_)_4_ and 0.2-Na_2_Pt(OH)_6_ show slightly lower and comparable activity to that of the 0.2-H_2_PtCl_6_ benchmark, respectively, which is comparable to previously reported CPA-derived catalysts.^36^ The similar performance is in line with the analogous octahedral geometry of the Na_2_Pt(OH)_6_ and H_2_PtCl_6_ precursors, which, even upon partial ligand depletion following deposition on the carbon support or under reaction temperature can lead to a nonplanar geometry that plausibly hinders reactant adsorption due to steric effects. Likewise, although undergoing hydrolysis in aqueous solutions before deposition on the carbon, Pt(NO_3_)_4_ is likely to also yield a nonplanar Pt site geometry.^37^ Conversely, 0.2-K_2_PtCl_4_ exhibits higher activity than 0.2-H_2_PtCl_6_, attributable to the square-planar configuration of the former Pt precursor with easier reactant access and activation instead of the octahedral one of CPA. All tetraammine-derived Pt SACs show improved performance by 70%, which could be linked to the square-planar geometry of their precursors. Only 0.2-[(NH_3_)_4_Pt]citrate shows minor activity improvement with respect to 0.2-H_2_PtCl_6_. This might be due to the reducing nature of the citrate counterion, which may affect the Pt sites or the neighboring functionalities in the carbon support and, in turn, reduce their respective hydrogen chloride and acetylene binding abilities. The superior initial activity of 0.2-[(NH_3_)_4_Pt]SO_4_, 0.2-[(NH_3_)_4_Pt](HCO_3_)_2_, and 0.2-[(NH_3_)_4_Pt]Cl_2_, compared with 0.2-H_2_PtCl_6_ is confirmed and even enhanced at 433 K, as the metal–ligand architecture in the Pt precursor is likely better preserved at lower temperature.

The ligand type of the Pt site, together with its geometry and electronic structure, is expected to play a central role in regulating reactant adsorption. To explore this, we conduct DFT simulations on different Pt*L*_2_ species (*L* = NH_3_, OH, NO_3_, and Cl) stabilized over O-functionalities in the AC support. These are modeled as tetraketone (keto_4_) sites (Figures 5b and S8, Table S6) in agreement with XPS results (*vide supra*, Figure S7; Tables S4–S7), as previous studies showed them to be most likely.^17^ While less abundant, Pt*L*_2_ species coordinated to S-functionalities are also modeled, as triketone-thiophene (keto_3_-S) and thiophene (S) sites (Table S7), for comparison. The initial activity of the Pt SACs correlates with the Gibbs energy of hydrogen chloride binding of the respective Pt*L*_2_ species, regardless of the coordinating functionality type (O or S) in the support. Specifically, Pt(NH_3_)_2_ species tend to bind hydrogen chloride (−0.59 eV) more easily than their PtCl_2_, Pt(NO_3_)_2_, and Pt(OH)_2_ counterparts (0.08, –0.32, and −0.35 eV, respectively), resulting in higher VCM productivity (Figure 5a). This could be explained by the softness of the ammine ligands compared to the hardness of hydroxyl, nitrate, and chloride ones: lesser electron donation from the ligands to the metal atom can favor hydrogen chloride binding, through interaction with the lone electron pairs of the chloride ion.

### Dynamics of Platinum Sites in Acetylene Hydrochlorination

**Figure 6:**
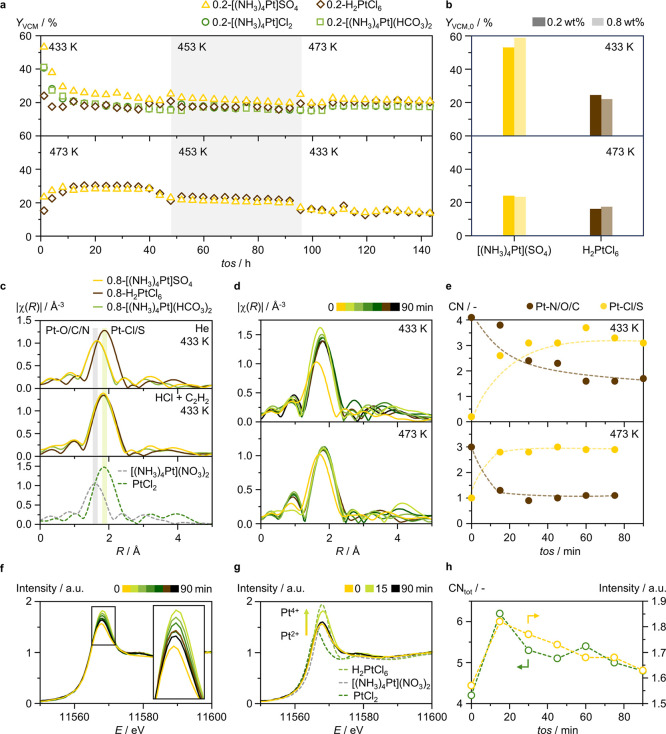
(a) Time-on-stream (*tos*) performance, expressed as *Y*_VCM_, of 0.2-[(NH_3_)_4_Pt]SO_4_, 0.2-[(NH_3_)_4_Pt](HCO_3_)_2_, and 0.2-[(NH_3_)_4_Pt]Cl_2_, compared with that of the 0.2-H_2_PtCl_6_ benchmark. (b) Initial *Y*_VCM_, *Y*_VCM,0_, over selected Pt SACs with 0.2 wt %, and 0.8 wt % metal content. The catalyst mass, and thus space velocity, was varied to maintain a constant reactant flow rate per metal site. Reaction conditions: *GHSV*(C_2_H_2_) = 325 h^–1^ (0.2 wt % Pt SACs, *W*_cat_ = 0.25 g), and 1300 h^–1^ (0.8 wt % Pt SACs, *W*_cat_ = 0.06 g), C_2_H_2_:HCl:Ar = 40:44:16, *T* = 433–473 K, *P* = 1 bar. (c) *Operando* Pt *L*_3_ edge EXAFS of the 0.8-[(NH_3_)_4_Pt]SO_4_, 0.8-[(NH_3_)_4_Pt](HCO_3_)_2_, and 0.8-H_2_PtCl_6_, together with *ex situ* spectra of reference compounds in dotted lines. (d) *Operando* Pt *L*_3_ edge EXAFS of 0.8-[(NH_3_)_4_Pt]SO_4_ at 433 and 473 K, together with (e) the corresponding coordination numbers as a function of *tos*. (f) *Operando* Pt *L*_3_ edge XANES of 0.8-[(NH_3_)_4_Pt]SO_4_ at 433 K, together with (g) *ex situ* spectra of reference compounds in dotted lines. (h) The total coordination number, summing Pt–Cl/S and Pt–O/C/N contributions, as a function of time on stream.

The robustness of the best-performing Pt SACs and the H_2_PtCl_6_-derived benchmark is assessed by evaluating them in acetylene hydrochlorination for 144 h on stream with increasing conversion, applying a *GHSV*(C_2_H_2_) of 325 h^–1^(Figure 6a). The catalytic test begins at 433 K, when the initial activity is the highest for the Pt SACs with ammine ligands, then the temperature is increased by 20 K every 48 h. While 0.2-[(NH_3_)_4_Pt]SO_4_, 0.2-[(NH_3_)_4_Pt](HCO_3_)_2_, and 0.2-[(NH_3_)_4_Pt]Cl_2_ initially exhibit a 2-fold enhanced VCM productivity compared with 0.2-H_2_PtCl_6_, their performance gradually converges to that of 0.2-H_2_PtCl_6_ within the first ca. 20 h on stream (Figure 6a). The catalytic test begins at 433 K, where Pt SACs with ammine ligands show the highest initial activity, exhibiting double the VCM productivity compared to 0.2-H_2_PtCl_6_. Over the first 20 h on stream, their performance gradually converges to the stable one of the 0.2-H_2_PtCl_6_ benchmark. Increasing the temperature by 20 K every 48 h reveals only slight increases in VCM yield. Furthermore, in agreement with the stable performance, minor coke formation is detected by thermogravimetric analysis (Table S10). When initiating the catalytic test at 473 K, performance convergence occurs faster (within ca. 10 h) followed by stable catalytic behavior, which is preserved upon reducing the reaction temperature in similar steps (Figure 6a). The initial convergence of the activity, followed by stable VCM productivity regardless of the platinum precursor used during the synthesis, points to the evolution of metal local environments to an identical structure. This process is accelerated by higher temperatures. The relatively small increase in VCM productivity upon ramping the temperature up could indicate that increasing the temperature causes further restructuring of the metal sites. This differs from the larger drops in VCM yield observed when decreasing the reaction temperature, which follows the expected temperature dependence, suggesting that the metal site structure is preserved.

*Operando* XAS analysis provides precise information on the underlying dynamic behavior of the Pt SACs derived from ammine-containing platinum precursors. For this purpose, we employ 0.8 wt % Pt SACs to enhance the quality of the collected spectra, as they exhibit similar catalytic behavior to the 0.2 wt % Pt SACs (Figure 6b). At 433 K in an inert atmosphere (He), 0.8-[(NH_3_)_4_Pt]SO_4_ and 0.8-[(NH_3_)_4_Pt](HCO_3_)_2_ present similar metal coordination environments (Figure 6c, Table S11). EXAFS analysis reveals a prominent Pt–O/C/N contribution (CN = 3.9 and 3.8, respectively), resulting from coordination with ammine ligands and O-functionalities in AC, and a minor Pt–Cl/S one (CN = 0.4 for both catalysts), linked to coordination to S-functionalities in the support. In contrast, the 0.8-H_2_PtCl_6_ shows a prominent Pt–Cl/S contribution (CN = 3.6), reflecting a high metal chlorination degree, and a smaller Pt–O/C/N contribution (CN = 0.7), corresponding to anchoring on AC. Under reaction conditions, both 0.8-[(NH_3_)_4_Pt]SO_4_ and 0.8-[(NH_3_)_4_Pt](HCO_3_)_2_ undergo chlorination, resulting in Pt–Cl/S spectral contributions (CN = 3.4 and 3.1, respectively) similar to 0.8-H_2_PtCl_6_. This explains the performance convergence between the tetraammine-derived and the CPA-derived Pt SACs over time (Figure 6a), as the former progressively reaches an equilibrium state of PtCl*_x_* (*x* = 2–3), similar to the latter. The 0.8-H_2_PtCl_6_ remains virtually unaltered, matching its stable catalytic performance (Figure 6a). No metal-acetylene interactions are observed in any Pt SACs, as the Pt–O/C/N contribution does not increase under reaction conditions (Table S10). Further time-resolved EXAFS analysis of 0.8-[(NH_3_)_4_Pt]SO_4_ under reaction conditions at 433 and 473 K shows that the Pt–Cl/S contribution increases while the Pt–N/O/C decreases (Figure 6d,e, Tables S12 and S13). This indicates an ammine-to-chloride ligand-exchange process that is faster at higher reaction temperature. This agrees with the more rapid convergence in performance between the tetraammine-derived Pt SACs and the CPA-derived Pt SAC observed at 473 K compared with 433 K (Figure 6a).

Notably, the metal chlorination process undergone by tetraammine-derived Pt SACs is reflected in a nonmonotone behavior in the XANES spectra of the working 0.8-[(NH_3_)_4_Pt]SO_4_ catalyst (Figure 6f). Under He, the Pt single atoms show similar spectral features to the [(NH_3_)_4_Pt](NO_3_)_2_ reference, indicating a Pt^2+^ oxidation state. Upon exposure to reaction conditions, we observe a sudden increase in the intensity and a slight shift to higher energy of the whiteline, resembling more the spectral features of the H_2_PtCl_6_ reference (Figure 6g). This is followed by a gradual loss in the intensity and a slight shift to lower energy of the whiteline over time on stream, resembling the PtCl_2_ reference. Such behavior reflects changes in the electronic configuration of the Pt species, which can relate to both or either the formal oxidation state (e.g., from Pt^2+^ to Pt^4+^ or vice versa) and the coordination environment (e.g., ammine-to-chloride ligand exchange, vide infra).^35,38,39^ The sudden increase in the whiteline intensity may be explained by the oxidative addition of HCl to Pt species, transitioning from Pt^2+^ to Pt^4+^. However, due to the lack of suitable reference materials with well-defined electronic configuration, assigning a formal oxidation state is tentative. Importantly, this potential initial change in the oxidation state of the Pt atoms, before stabilization as PtCl*_x_* (*x* = 2–3) species, does not seem necessary for fulfilling the catalytic cycle and would rather reflect the ammine-to-chloride ligand exchange process. This is further supported by the absence of appreciable alterations in the XANES features of 0.8-H_2_PtCl_6_ in acetylene hydrochlorination, where the PtCl*_x_* (*x* = 2–3) species undergo slight chlorination reflecting HCl-binding (Figure S9, Tables S14 and S15). The nonmonotone XANES behavior of 0.8-[(NH_3_)_4_Pt]SO_4_ under reaction conditions can be correlated with the total coordination number of the Pt species, accounting for both Pt–O/C/N and Pt–Cl/S contributions (Figure 6h). EXAFS analysis of the individual contributions suggests that the Pt atoms bind hydrogen chloride at a faster rate than ammine-ligand depletion (Figure 6e). Therefore, the sudden increase in whiteline intensity can be correlated with the total Pt coordination, including ammine ligands and the rapidly increasing chloride ligands, reflecting high hydrogen chloride activation and high initial activity. The progressive reduction in whiteline intensity could be linked to the gradual loss of ammine ligands, resulting in performance convergence of the tetraammine-derived Pt SACs to that of the CPA-derived counterpart (Figure 6a).

**Figure 7:**
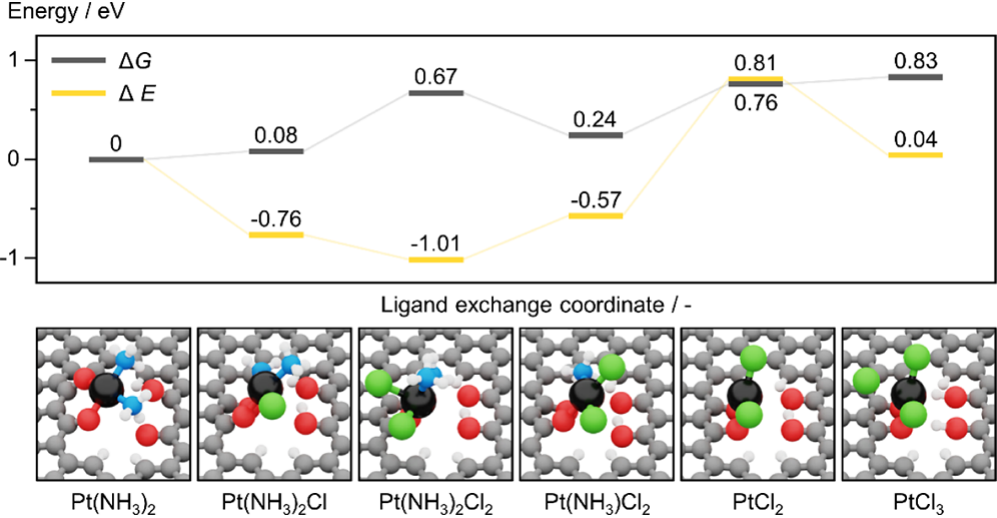
Gibbs free energy, Δ*G*, and potential energy, Δ*E*, of the progressive chloride-ammonia ligand exchange that Pt SACs undergo, herein investigated for Pt(NH_3_)_2_ species anchored over O-functionalities, keto_4_, in AC, as illustrated by schematic representations (top). *E* was calculated in reference to HCl and NH_3_ molecules in the gas phase. *G* accounts for the gas-phase entropy of HCl and NH_3_ molecules.

Guided by the structural information on the working Pt atoms provided by *operando* XAS, we employ DFT simulations to gain mechanistic insights into the ammine-chloride ligand exchange process (Figure 7). Consistent with experimental observations of rapid chlorination (Figure 6e), Pt(NH_3_)_2_ species, stabilized on keto_4_ functionalities in AC, can dissociatively activate two hydrogen chloride molecules by coordinating the chloride ions while the proton transfers to the support (Gibbs energy, Δ*G* = 0.08 and 0.67 eV, respectively). The subsequent loss of one ammine ligand is exergonic, while the depletion of the second ammine ligand is also energetically accessible (Δ*G* = 0.24 and 0.76 eV, respectively). Lastly, further chlorination is energetically virtually neutral (Δ*G* = 0.83 eV), pointing to the coexistence of PtCl_2_ and PtCl_3_ species.^40^

### Reaction Mechanism and Kinetic Modeling

Monitoring the dynamic behavior of working AC-supported Pt atoms in acetylene hydrochlorination via XAS does not evidence any metal-acetylene interaction, regardless of the specific metal–ligand structures. Conversely, only Cl-binding is observed, reflecting the high affinity of Pt atoms for HCl regardless of their ligand and support environment as corroborated computationally (Table S16). This is in line with previous investigations, evidencing the bifunctional role of Pt atoms, activating hydrogen chloride, and neighboring carbon functionalities, binding acetylene, in fulfilling the catalytic cycle.^17^ Accordingly, XPS analysis of 0.8-[(NH_3_)_4_Pt]SO_4_ after reaction shows chlorination of the Pt atoms, as well as the AC support, but also an alteration in the surface O-functionalities, which is attributable to interaction with acetylene (Figure S7, Table S6). DFT simulations exploring competitive adsorption between hydrogen chloride and acetylene further corroborate the stronger interaction of Pt single atoms for the former, preferred to the latter by up to −1.65 eV (Table S17). Conversely, acetylene can adsorb readily on metal-neighboring O-functionalities in the carbon support, irrespective of the metal–ligand architecture, by forming five- or six-membered rings (Table S18).^17^ While XAS, XPS, and DFT analyses provide valuable insights into the active site dynamics and the reaction mechanism, kinetic investigations are necessary to understand the influence of operating variables such as pressure, temperature, and space-velocity on reaction rates. This serves as a basis for modeling and predicting the macroscopic catalytic behavior, which constitutes a critical aspect of process scale-up studies.

**Figure 8:**
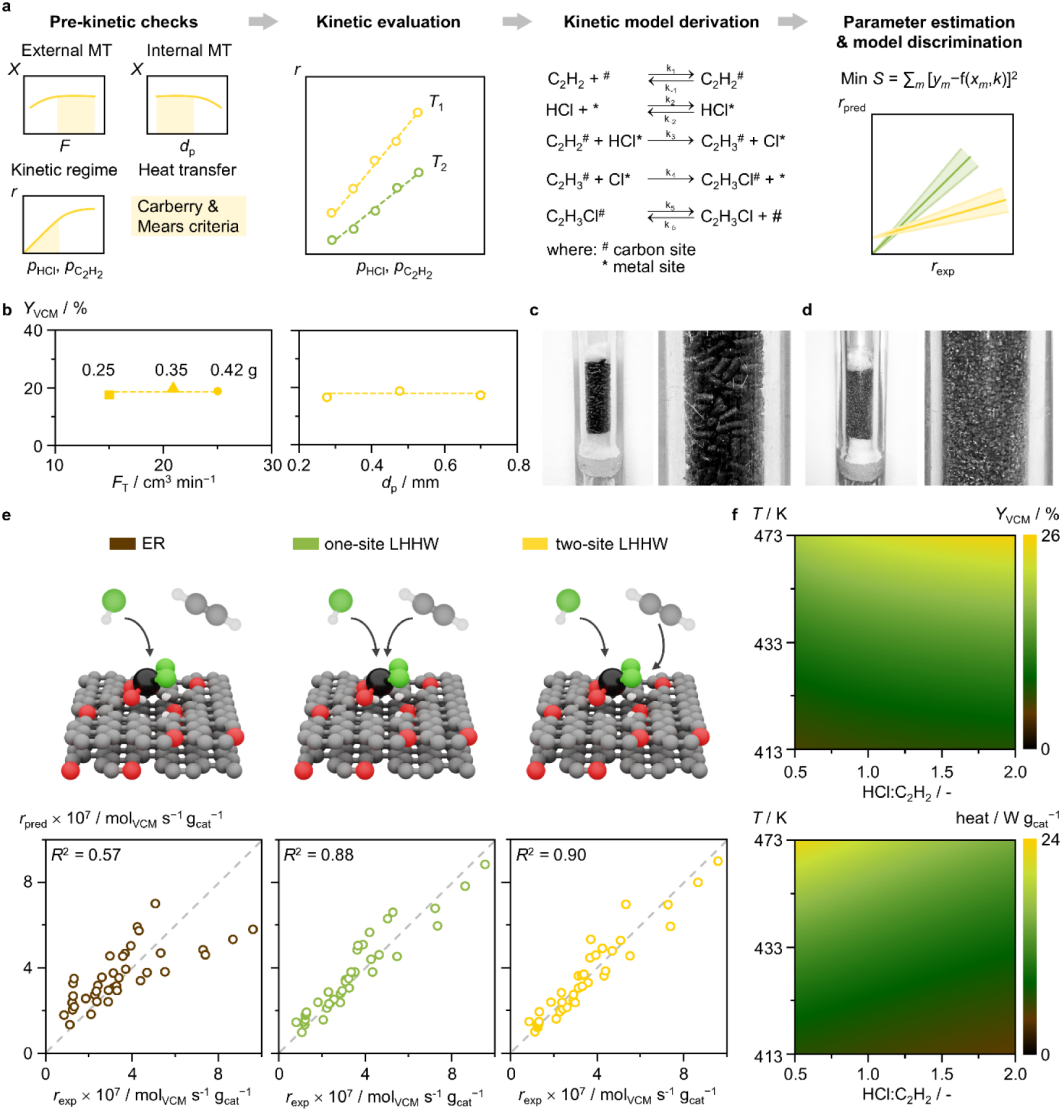
(a) Schematic representation of the steps followed in modeling the kinetics of acetylene hydrochlorination over Pt SACs. (b) Assessment of the absence of extra-particle (left) and intraparticle (right) mass-transfer limitations over the 0.2-[(NH_3_)_4_Pt]SO_4_, upon equilibration for 48 h at 473 K in acetylene hydrochlorination conditions, under variable flow rates and constant space velocity (left) and variable catalyst particle size and constant flow rates (right). Reaction conditions: *F*_T_/*W*_cat_ = 3600 cm^3^ min^–1^ g_cat_^–1^, C_2_H_2_:HCl:Ar = 40:44:16, *T* = 473 K, *P* = 1 bar. (c,d) Photographs of packed bed reactor with catalyst in extrudate and sieved particle (0.4–0.6 mm) forms, respectively. (e) Schematic representation of the analyzed potential reaction mechanisms (top) with parity plots of the respective developed kinetic models (bottom). (f) *Y*_VCM_ (top) and heat of reaction (bottom) as a function of reaction temperature and reactant ratio, as predicted by the two-site LHHW model.

A series of steps are followed to model the kinetics of acetylene hydrochlorination over 0.2-[(NH_3_)_4_Pt]SO_4_ (Figure 8a), upon equilibration under reaction conditions at 473 K for 48 h. Catalytic tests performed (i) at variable flow rates and constant *GHSV*(C_2_H_2_) and (ii) at constant *GHSV*(C_2_H_2_) using the catalyst in the form of extrudates as well as sieved particles of different sizes respectively corroborated the absence of extra- and intraparticle mass transfer limitations (Figure 8b, Table S19). This result exemplifies the possibility for academic groups to work not only with small catalyst particles but also industrially relevant bodies such as extrudates (Figure 8c,d), without leading to loss in performance. Thereafter, the dependence of the overall reaction rate on the partial pressure of each reactant is investigated to identify the region of kinetic control (Figure S10). Partial reaction orders with respect to hydrogen chloride and acetylene are determined (0.37 and 0.86, respectively, Figure S11). The measured partial reaction orders suggest that hydrogen chloride adsorption is fast, pointing to the elementary steps involving acetylene being kinetically limited.^41^ The obtained values, lower than unity, suggest participation in the catalytic cycle as chemisorbed species. Notably, the apparent activation energy of 0.37 eV (Figure S11) aligns with the value of 0.51 eV that we recently calculated by DFT simulations for a bifunctional mechanism over bichlorinated Pt atoms and neighboring sites in the carbon.^17^

Based on the XAS-resolved steady-state PtCl*_x_* (*x* = 2–3) active species (Figure 6e), we investigate possible reaction mechanisms of acetylene hydrochlorination previously reported in the literature.^15,17,42^ For this purpose, we evaluate three kinetic models based on (i) an Eley–Rideal (ER) mechanism, wherein hydrogen chloride and acetylene respectively chemisorb and physisorb on the metal site; (ii) a one-site Langmuir–Hinshelwood–Hougen–Watson (LHHW) mechanism, wherein hydrogen chloride and acetylene both chemisorb on the metal site; and (iii) a two-site LHHW mechanism, wherein the hydrogen chloride chemisorbs on the metal site while acetylene chemisorbs on a neighboring site in the carbon support (Figure 8e, Table S20). We note that the ER and LHHW models are derived accounting for the isolated nature of the Pt atoms, by carefully adapting the elementary steps similarly to mechanistic studies in homogeneous catalysis.^43^ In all cases, the rate-determining step is assumed to be the H-addition for VCM formation. Following the determination of kinetic parameters for each model by fitting model predictions with experimentally measured rates, the outcomes of the three models are compared using parity plots (Figure 8e). At first glance, the ER model displays a suboptimal fit, displaying a coefficient of determination of 0.57. Furthermore, the equilibrium constant for hydrogen chloride adsorption is calculated to be much lower than unity (0.37 bar^–1^), contrasting with the ease of hydrogen chloride binding to Pt atoms observed in spectroscopic and computational analyses. These observations indicate that ER is not the leading mechanism for the acetylene hydrochlorination reaction over Pt SACs. Conversely, both the one- and two-site LHHW models present high agreement between experimental and predicted VCM rates, respectively exhibiting a coefficient of determination of 0.88 and 0.90. In both LHHW models the equilibrium constants determined for the adsorption of hydrogen chloride and acetylene show the former being one-order-of-magnitude higher than the latter. This suggests a stronger affinity of the catalyst surface for hydrogen chloride compared to acetylene, as also indicated by their respective partial reaction orders. While these results point to the participation of both reactants in the catalytic cycle as chemisorbed species, the two-site LHHW model agrees with the active site structure uncovered by spectroscopic and computational analyses (*vide supra*), comprising both Pt atoms and neighboring carbon functionalities respectively chemisorbing hydrogen chloride and acetylene.

The identification of the reaction mechanism and the development of a microkinetic model are necessary to predict conditions for optimal reactor operation. Any suitable drop-in replacement for the industrial Hg-based catalysts requires a low light-off temperature conducive to catalyst startup, within the temperature range available in the existing reactor infrastructure, for effective management of the exothermicity of acetylene hydrochlorination under operation.^44^ In the case of the Hg- and Au-based catalysts, the formation of hotspots leads to deactivation, by metal volatilization and reduction, respectively.^11^ The VCM yield and the reaction heat of a representative catalyst, 0.2-[(NH_3_)_4_Pt]SO_4_ upon equilibration, are predicted using the two-site LHHW model as a function of temperature and reactant concentrations. Since reaction light-off commonly occurs between 413 and 473 K,^15,44^ this temperature range is selected. Importantly, this analysis shows that Pt SACs exhibit a low light-off temperature (Figure 8f), around 413 K at equimolar reactant ratio, which is further reduced in as-prepared Pt SACs derived from ammine-based metal precursors (Figure 5). Elevated temperatures would lead to high VCM yield while variations in the reactant ratio would not significantly influence it. Nevertheless, elevated temperatures and low hydrogen chloride-to-acetylene ratio would also result in high overall reaction rate and thus heat production (Figure 8f). Correct heat management by appropriate cooling systems, e.g., steam-raising, routinely implemented in acetylene hydrochlorination reactors, would enable maximized productivity and avoidance of hot spots. In future efforts, catalyst testing in a larger pilot-type reactor would provide detailed insights into the exotherm region and axial temperature profile that Pt SACs would exhibit in industrial tubular fixed-bed reactors.

### Toxicity Assessment of Platinum Precursors

**Figure 9:**
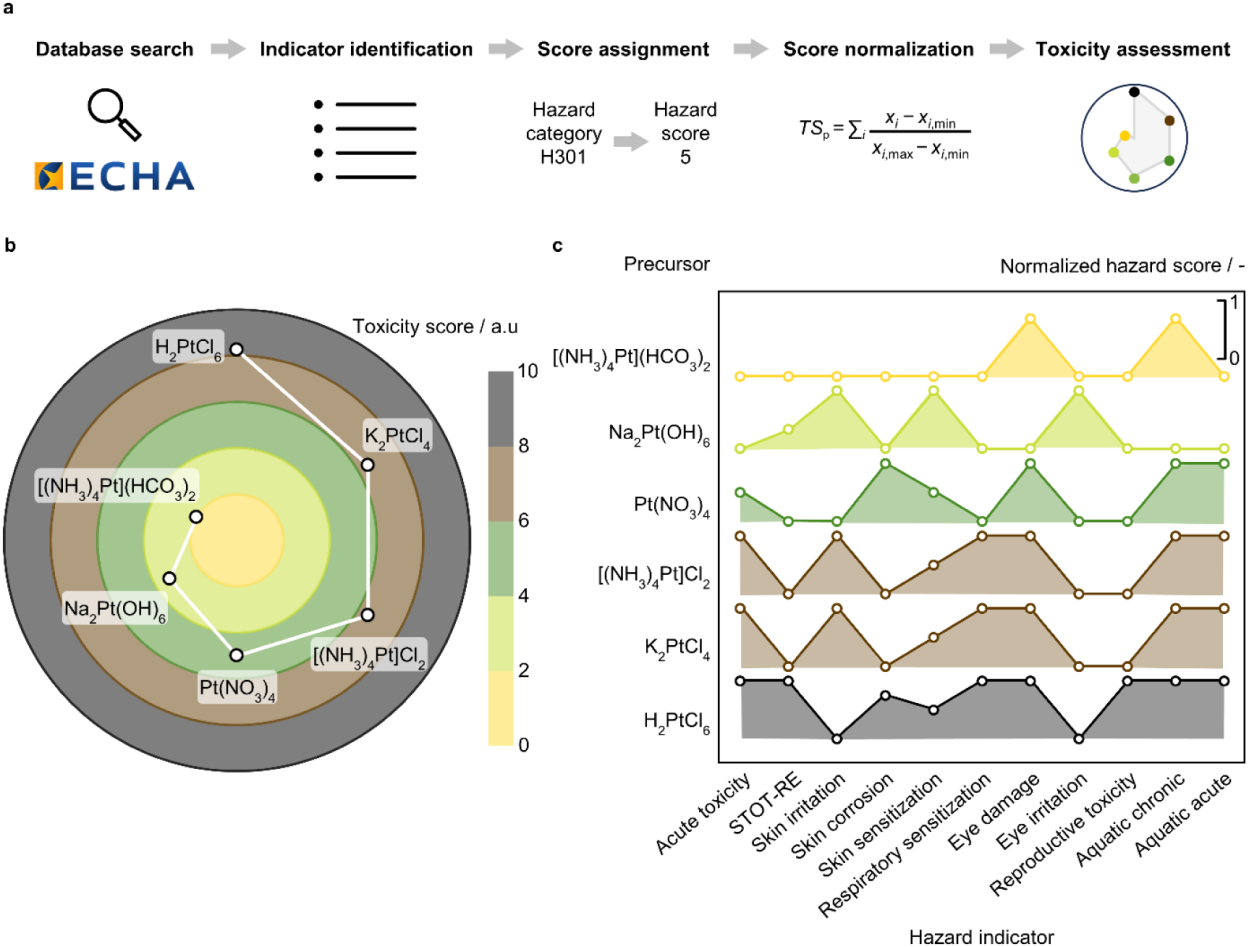
(a) Schematic representation of the multiparameter approach developed to assess the toxicity of metal compounds. (b) Toxicity score of the Pt precursors, and (c) the individual contribution of each hazard indicator, wherein a normalized hazard score of zero indicates that the Pt precursor attains the minimum (best) score for a given hazard category across all Pt precursors, while one corresponds to the maximum (worst) score. In (c), STOT-RE stands for specific target organ toxicity – repeated exposure.

Assessing the toxicity of metal precursors is key for ensuring safety and minimizing environmental impact in Pt SACs manufacture and eventual commercialization. The European Chemicals Agency (ECHA) establishes procedures for assessing and documenting hazards of substances through the Registration, Evaluation, Authorisation, and Restriction of Chemicals (REACH) and the Classification, Labeling, and Packaging (CLP) regulations.^28^ The latter standardizes hazard classification via pictograms and hazard codes. These are based on quantitative data, such as the median lethal dose, as well as observed effects collected through available experimental assessments. Nevertheless, there is a lack of universally agreed aggregated metrics evaluating substance toxicity to human health and the environment. Considering these two domains, we develop a methodology that assigns a numerical value to the CLP hazard codes associated with each precursor based on the hazard severity (Figure 9a). Normalization and aggregation yield a toxicity score for the platinum precursors, enabling a quantitative comparison. Physical hazards to equipment (e.g., metal corrosion) are not considered in the assessment provided mitigation measures are available (e.g., corrosion-resistant materials). The [(NH_3_)_4_Pt]SO_4_ and [(NH_3_)_4_Pt]citrate precursors, custom-made for this study, are not documented in the ECHA database, and therefore are omitted from this analysis.

While platinum precursors inherently exhibit hazardous properties owing to the platinum cation itself, their chemical structure greatly influences the nature and extent (Figure 9b,c).^45^ In line with its pH-neutral nature and nonoxidizing ligands and counterions, the [(NH_3_)_4_Pt](HCO_3_)_2_ precursor exhibits the lowest toxicity score (2.0), followed by the Na_2_Pt(OH)_6_ precursor (3.3) exhibiting skin irritation and sensitization effects, among others, owing to the basic properties of the hydroxyl groups. The Pt(NO_3_)_4_, [(NH_3_)_4_Pt]Cl_2_, and K_2_PtCl_4_ precursors present similar toxicity scores (5.0, 6.5, and 6.5, respectively). Regarding the Pt(NO_3_)_4_ compound, its skin-corrosive properties together with the harm to aquatic environments negatively impact this metric. Nevertheless, the dissolution of Pt(NO_3_)_4_ in water, an essential step in the Pt SAC synthesis method via impregnation herein developed, likely leads to nitrate-water ligand exchange resulting in a Pt complex with reduced toxicity than undissolved Pt(NO_3_)_4_. Compounds containing chloride ions, either in the form of ligands or counterions, manifest acute toxicity as well as irritation and sensitization of skin, eyes, and respiratory system. This is attributable to the water solubility that metal-chloride compounds present, reflecting high solubility in biofluids, coupled with proneness to hydrolysis and release of dangerous compounds (e.g., HCl).^29^ At last, and consistent with its chlorinated and acidic nature, H_2_PtCl_6_ is identified as the most hazardous precursor, with a toxicity score of 8.3, showing signs of substantial potential for damage to human health and the environment.

In addition to the intrinsic toxicity of the metal precursors, significant risks may arise from accidental mixing with other chemicals in laboratory or industrial environments. To evaluate the risk associated with the reactivity of the platinum precursors with common chemicals, we employ the Chemical Reactivity Worksheet (CRW4) software,^29^ developed by the American National Oceanic and Atmospheric Administration and Institute of Chemical Engineers (NOAA and AIChE, respectively). This tool predicts the potential hazards of chemical mixtures based on experimental data and previous incidents. An overall reactivity score, based on 14 common chemicals, is obtained for each Pt precursor following a similar approach to that developed to evaluate their toxicity score: considering the minimum and maximum reactivity scores across Pt precursors, with subsequent normalization and aggregation into a single metric (Figures S12 and S13). In line with the pH-neutral properties and the moderate reactivity of both NH_3_ ligand and HCO_3_^–^ counterions, [(NH_3_)_4_Pt](HCO_3_)_2_ also exhibits the lowest reactivity score. As a result, [(NH_3_)_4_Pt](HCO_3_)_2_ emerges as the safest metal precursor for Pt SAC preparation from the hazard (i.e., toxicity and reactivity) assessment herein conducted. While these preliminary analyses indicate suitability for commercialization, more in-depth investigations would be required to more accurately identify the type and extent of hazards and, consequently, define mitigation measures.

## Conclusion

This study explored the impact of metal precursors on the development of Pt SACs for acetylene hydrochlorination aiming to identify potential benefits for the synthesis or performance compared with the typically employed CPA. For this purpose, we investigated a series of Pt^2+^ and Pt^4+^ customized complexes featuring ammine, hydroxyl, nitrate, and chloride ligands. To enhance the practical scope of this catalytic technology, we employed a standardized and scalable impregnation protocol on activated carbon extrudates. XAS analysis showed that the supported Pt single atoms are stabilized on the support surface in a Pt^2+^ oxidation state regardless of the electronic state in the respective metal precursor. Nevertheless, the different metal–ligand architectures exhibited distinct initial activities, correlating with their hydrogen-chloride binding ability as indicated by DFT simulations. Pt SACs obtained from tetraammine precursors demonstrated up to a 2-fold higher initial activity than their CPA-derived counterparts. Their performance gradually converged to the same VCM productivity due to dynamic restructuring, while maintaining outstanding precursor-independent stability over 150 h. This catalytic behavior is linked by combined *operando* XAS and DFT analyses to a reaction-induced formation of common, active and stable Pt–Cl*_x_* (*x* = 2–3) species, via a ligand-chloride exchange process. Based on the identified steady-state active site structure, we developed a LHHW kinetic model bridging atomic-scale reaction pathways with macroscopic catalytic behavior, additionally providing predictive information to facilitate process implementation. Most importantly, the convergent active site evolution in Pt SACs and the related precursor-independent robustness enable toxicity minimization through multiparameter assessment, favoring halide-free and pH-neutral Pt complexes. Beyond acetylene hydrochlorination, our work underscores the importance of often-overlooked toxicity analyses in catalyst design programs and the flexibility in metal precursor selection that convergent active site evolution enables.

## Data Availability

The experimental and computational data sets presented in this study is open sourced at the Zenodo (https://doi.org/10.5281/zenodo.11179606) and ioChem BD (https://iochem-bd.iciq.es/browse/review-collection/100/69172/35d571e8b245d54e268cd6a9) databases,^46^ respectively.

## Supporting Information Available

Description of catalyst synthesis, characterization, and evaluation together with computational methods and toxicity analyses (PDF)
