# Supplementary material for: Convergent Active Site Evolution in Platinum Single Atom Catalysts for Acetylene Hydrochlorination and Implications for Toxicity Minimization
Source: ACS Catal. 2024 Aug 29;14(18):13652–64. doi: 10.1021/acscatal.4c03533 (PMC11420957; doi:10.1021/acscatal.4c03533)
Supplement: Supplementary file 1 — cs4c03533_si_001.pdf [file cs4c03533_si_001.pdf]

Supporting Information

Convergent Active Site Evolution in Platinum Single Atom Catalysts for Acetylene Hydrochlorination and Implications for Toxicity Minimization

*V. Giulimondi,[a] M. Vanni,[a] S. Damir,[a] T. Zou,[a] S. Mitchell,[a] F. Krumeich,[a] A. Ruiz-Ferrando,[b,c] N. López,[b] J.J. Gata-Cuesta,[a] G. Guillén-Gosálbez,[a] J.J. Smit,[d] P. Johnston,[e] and J. Pérez-Ramírez[a]**

[a] Department of Chemistry and Applied Biosciences, ETH Zürich, Vladimir-Prelog-Weg 1, 8093 Zürich, Switzerland.

[b] Institute of Chemical Research of Catalonia (ICIQ-CERCA), Av. Països Catalans 16, 43007 Tarragona, Spain.

[c] University of Rovira i Virgili, Av. Catalunya 35, 43002 Tarragona, Spain.

[d] Johnson Matthey, Catalyst Technologies, Eastbourne Terrace 10, W2 6LG London, U.K.

[e] Johnson Matthey, Catalyst Technologies, Belasis Avenue 1, TS23 1LB Billingham, U.K.

* Corresponding author: jpr@chem.ethz.ch.

Table of contents

1. Supporting experimental procedures 2

1.1. Catalyst preparation 2

1.2. Catalyst characterization 2

1.3. Catalytic evaluation 4

1.4. Evaluation of mass and heat transfer limitations 5

1.5. Kinetic modeling 6

1.5.1. Approach and notation 6

1.5.2. Derivation of the ER model 7

1.5.3. Derivation of the one-site LHHW model 8

1.5.4. Derivation of the two-site LHHW model 9

1.5.5. Overall rate calculation and parameter fitting 11

1.6. Computational methods 12

1.7. Evaluation of metal precursor toxicity and reactivity 12

2. Supporting tables 16

3. Supporting figures 36

4. Supporting references 49

1. Supplemental experimental procedures

1.1. Catalyst preparation

The platinum single atom catalysts (Pt SACs) were synthesized at Johnson Matthey facilities using a procedure following industrial catalyst manufacture standards. Specifically, the Pt SACs were prepared via incipient wetness impregnation using commercial activated carbon with a nominal metal content of 0.2 or 0.8 wt%. Activated carbon (AC) was procured from Norit (ROX 0.8), while H_2PtCl_6, K_2PtCl_4, $Na_2Pt(OH)_6$, $Pt(NO_3)_4$, $[(NH_3)_4Pt]Cl_2$, $[(NH_3)_4Pt]$citrate, and $[(NH_3)_4Pt](HCO_3)_2$ were custom-synthesized, and H_2SO_4 was purchased from Fisher Scientific. The as-received AC extrudates were dried at 378 K in air for 24 h. The impregnation was conducted at full pore volume. H_2PtCl_6 was dissolved in water (53 cm^3) at ambient temperature. The solution was added dropwise to the AC extrudates (50 g) with agitation and mixing. The obtained materials were allowed to stand for *ca.* 30 min, after which there was no excess or surface solution, and subsequently transferred to a drying dish and dried at 383 K in air for 16 h. The synthesis procedure with K_2PtCl_4, $Na_2Pt(OH)_6$, $Pt(NO_3)_4$, $[(NH_3)_4Pt]Cl_2$, and $[(NH_3)_4Pt]$citrate was equivalent to the one described above. The catalysts derived from $[(NH_3)_4Pt](HCO_3)_2$ and $[(NH_3)_4Pt]SO_4$ were prepared by dissolving $[(NH_3)_4Pt](HCO_3)_2$ in hot water (53 cm^3), and in the case of $[(NH_3)_4Pt]SO_4$ by adding H_2SO_4 until pH 3.6 was reached. All other steps were unchanged. All obtained Pt SACs are denoted as **X-M** (metal content, X = 0.2 or 0.8; metal precursor, M), wherein the AC support is omitted for simplicity.

1.2. Catalyst characterization

Elemental composition analysis of AC (C, H, N, O, S, Cl) was conducted using a LECO CHN-900 elemental analyzer equipped with an infrared spectrometer.

Inductively coupled plasma optical emission spectrometry (ICP-OES) was conducted using a Horiba Ultra 2 instrument equipped with a photomultiplier tube detector. The sample was dissolved in an Anton-Paar Multiwave 7000 microwave digestion system, using a 1:4 mixture of HCl (VWR Chemicals, 37%) and HNO_3 (Sigma-Aldrich, 65%) at 513 K and 80 bar of Ar.

Nitrogen sorption isotherms were measured at 77 K in a Micromeritics Tristar II, after degassing the solids at 473 K for 12 h.

Mercury porosimetry was measured at 293 K in a Micromeritics AutoPore 9500 porosimeter operated from vacuum to 224 MPa. A contact angle of 130° for mercury was applied.

Micro-computed tomography (Micro-CT) analysis was performed using an EasyTom XL Ultra 230-160 micro/nano-CT scanner (RX Solutions). The scanner was operated at 45 kV and a current of 180 μA. The sample was scanned over a full 360° with a rotation step of 0.15°. A CCD camera detector was employed, with exposure time of 2 s and frame average of 3. The nominal resolution was set to 250 nm voxel size. Scan images were reconstructed using the XAct (RX Solutions) software with two different window widths to highlight differences in the radiodensity within the analyzed material. The resulting tomographic slices (16-bit TIFF format) have a size of 4086x3641 voxels (2250 images per

dataset) and a voxel size of 250 nm. Three-dimensional reconstructions of micro-CT slices were generated using Amira software.

Scanning electron microscopy with back-scattered electrons (BSE-SEM) imaging and energy dispersive X-ray (EDX) maps were acquired on a SEM FEI Quanta 200F operated at 30 kV. The samples were embedded in epoxydic resin using a Struers CitoVac cold embedding unit and mechanically polished on a Struers Tegramin to expose flat cross-sections of the extrudates. A thin carbon coating (5 nm) was applied before the measurement.

Powder X-ray diffraction (XRD) was measured using a PANalytical X'Pert PRO-MPD diffractometer with Cu-Kα radiation (λ = 1.54060 Å). The data was recorded in the 10-70° 2θ range with an angular step size of 0.017° and a counting time of 0.26 s per step.

Scanning transmission electron micrographs (STEM) with a high-angle annular dark-field (HAADF) detector were acquired on an aberration-corrected HD2700CS (Hitachi) and JEOL ARM-200F microscopes operated at 200 kV. Samples were prepared by dipping the copper grid supporting a holey carbon foil in a suspension of the solid in ethanol and drying in air.

X-ray photoelectron spectroscopy (XPS) measurements were conducted on a Physical Electronics Quantum 2000 instrument using monochromatic Al-Kα radiation, generated from an electron beam operated at 15 kV, and equipped with a hemispherical capacitor electron-energy analyzer. The samples were analyzed at an electron take-off angle of 45° and a constant analyzer pass energy of 46.95 eV with a spectra resolution step width of 0.2 eV. The spectrometer was calibrated for the Au 4*f* 7/2 signal at 84.0±0.1 eV. The Pt 4*f*, Cl 2*p*, O 1*s*, and S 2*p* spectra were fitted after Shirley background subtraction. The selected peak positions are based on literature data.[1-8]

X-ray absorption spectroscopy (XAS) measurements were performed at the Swiss-Norwegian beamlines (SNBL, BM31) at the European Synchrotron Radiation Facility (ESRF).[9] The X-ray beam was collimated using a double-crystal liquid nitrogen-cooled Si(111) monochromator and calibrated using Pt foil.[9] The gas filling inside the ionization chambers ensured optimal absorption at the Pt L_3 absorption edge. Transmission and fluorescence geometry configurations were used for the analysis of Pt in *operando* and *ex situ* modes, respectively, using a one-element silicon drift detector with Peltier cooling. Continuous scanning was performed for the Pt L_3 edge (between 11.45 and 12.10 keV), and the step size was set to 0.6 eV, with a scan duration of 180 s. The incident X-ray beam was focused on a 3 mm (horizontal) by 0.2 mm (vertical) area. Provided the corrosiveness of HCl, an experimental set-up, described elsewhere,[10] was designed and employed for conducting XAS measurements in *operando* mode while ensuring the equipment and personnel safety. For this latter purpose, the gases C_2H_2 (Air Liquide, purity 2.5), HCl (Air Liquide, 1 vol% in He, anhydrous), Ar (Air Products, BIP®, internal standard), and He (Air Products, BIP®, carrier gas) were fed to ensure that the HCl concentration would not exceed 1 vol.%. The catalyst was placed between two plugs of quartz wool in a quartz capillary reactor cell (outer diameter = 2 mm, wall thickness = 0.01 mm). Prior to feeding the reactant mixtures (0.86 vol.% C_2H_2, 0.95 vol.% HCl, 1 vol.% Ar, 97.19 vol.% He) at a total volumetric flow rate of

95 cm^3 min^{-1}, the catalysts (m_{cat} = 13 mg) were heated under He flow to the desired temperature (433 or 473 K). XAS scans were collected during the whole process. The resulting spectra were energy calibrated, background corrected, normalized, and analyzed using the Demeter software package.[11] k^3-weighted extended X-ray absorption fine structure (EXAFS) spectra were fitted in the optimal k-space (3-8.7 Å^{-1}) and R-space (1.1-3 Å) windows. An amplitude reduction factor of 0.77 was determined by fitting the EXAFS spectrum of a Pt foil.

Thermogravimetric analysis (TGA) was performed using a Linseis STA PT1600 system. TGA of the as-prepared catalysts and after use in acetylene hydrochlorination was carried out in diluted oxygen (20 vol.% O_2/Ar, 100 cm^3 min^{-1}), heating the samples (amount fixed to 20 mg) from 298 to 1273 K at 10 K min^{-1}.

1.3. Catalytic evaluation

The hydrochlorination of acetylene to vinyl chloride (VCM) was evaluated at atmospheric pressure in a continuous-flow fixed-bed micro-reactor, described elsewhere.[1] The gases C_2H_2 (PanGas, purity 2.6), HCl (Air Liquide, purity 2.8, anhydrous), Ar (PanGas, purity 5.0, internal standard), and He (PanGas, purity 5.0, carrier gas), were fed using digital mass-flow controllers (Bronkhorst) to the mixing unit, equipped with a pressure indicator. A quartz micro-reactor of 8 mm internal diameter was loaded with the catalyst (m_{cat} = 0.25 g) and placed in a home-made electrical oven. A K-type thermocouple fixed in a co-axial quartz thermowell with the tip positioned in the center of the catalyst bed was used to control the temperature during the reaction. Prior to testing, the catalyst was heated in a He flow to the desired bed temperature (T = 433-473 K) and allowed to stabilize for at least 15 min before the reaction mixture (40 vol% C_2H_2, 44 vol% HCl, and 16 vol% Ar) was fed at a total volumetric flow rate of F_T = 7.5-15 cm^3 min^{-1}. Reaction kinetics of acetylene hydrochlorination was studied over the 0.2-[$(NH_3)_4$Pt]SO_4 catalyst upon stabilization for 48 h under the following reaction conditions: F_T = 15 cm^3 min^{-1}, 40 vol% C_2H_2, 44 vol% HCl, and 16 vol% Ar, m_{cat} = 0.33 g, T = 473 K. Thereafter, kinetic measurements were conducted in the temperature range of 413-473 K at conversion levels <20% with a catalyst mass of m_{cat} = 0.33 g, a total flow of F_T = 15 cm^3 min^{-1}, and reactant concentrations of 10-44 vol%, balanced in He. The kinetic data was employed to determine the apparent activation energy (E_a) and the partial reaction order of the reactants ($n_{C_2H_2}$, n_{HCl}), and as input data for modeling analysis (*vide infra*). Carbon-containing compounds (C_2H_2 and C_2H_3Cl) and Ar were quantified on-line via a gas chromatograph equipped with a GS-Carbon PLOT column coupled to a mass spectrometer (GC-MS, Agilent, GC 7890B, Agilent MSD 5977A). Since VCM was the only product detected in all our tests, the catalytic activity is presented as the yield of VCM, Y_{VCM}, calculated according to Eq. 1,

$$Y_{VCM},\% = \frac{n_{VCM}^{outlet}}{n_{C_2H_2}^{inlet}} \times 100 \qquad \text{Eq. 1}$$

where n_{VCM}^{outlet} and $n_{C_2H_2}^{inlet}$ denote the molar flows of VCM and C_2H_2 at the reactor outlet and inlet, respectively. The overall reaction rate (r) was determined according to Eq. 2,

$$r,\ \text{mol}_{\text{VCM}}\ \text{s}^{-1}\ \text{g}_{\text{cat}}^{-1} = \frac{n_{\text{VCM}}^{\text{outlet}}}{m_{\text{cat}}} \qquad \text{Eq. 2}$$

where m_{cat} denotes the catalyst mass. The turnover frequency, *TOF*, were determined according to Eq. 3,

$$TOF = \frac{n_{\text{VCM}}^{\text{outlet}}}{n_{\text{Pt}}} \qquad \text{Eq. 3}$$

where n_{Pt} denotes the amount of Pt moles. The error of the carbon balance, ε_{C}, determined using Eq. 4, was less than 10% in all experiments, i.e., the carbon mass balance was closed at ≥90%.

$$\varepsilon_{\text{C}},\% = \frac{n_{\text{C}_2\text{H}_2}^{\text{inlet}} - \left(n_{\text{C}_2\text{H}_2}^{\text{outlet}} + n_{\text{VCM}}^{\text{outlet}}\right)}{n_{\text{C}_2\text{H}_2}^{\text{inlet}}} \times 100 \qquad \text{Eq. 4}$$

After the tests, the reactor was quenched to room temperature in He flow and the catalyst was retrieved for further characterization.

1.4. Evaluation of mass and heat transfer limitations

The evaluation of the dimensionless moduli based on the criteria of Carberry,[12] Mears,[13] and Weisz-Prater,[14] confirmed that all the catalytic tests were performed in the absence of mass and heat transfer limitations. The Carberry criterion (*Ca*) was used to evaluate external mass transfer limitations according to Eq. 5,

$$Ca = \frac{r_{\text{v,obs}}}{a' k_{\text{f}} c_{\text{b}}} < \frac{0.05}{|n|} \qquad \text{Eq. 5}$$

where k_{f} is the mass transfer coefficient (estimated at a minimum value of 0.01 m s^{-1}), c_{b} is the bulk concentration of acetylene (17.6 mol m^{-3}), n is the reaction order, while $r_{\text{v,obs}}$ and a' denote the reaction rate and the specific particle area, which are derived via Eq. 6 and Eq. 7, respectively,

$$r_{\text{v,obs}} = \frac{n_{\text{VCM}}^{\text{outlet}}}{V_{\text{cat}}} \qquad \text{Eq. 6}$$

$$a' = \frac{1}{L} = \frac{A_{\text{p}}}{V_{\text{p}}} = \frac{4}{d_{\text{p}}} \qquad \text{Eq. 7}$$

where L, A_{p}, V_{p}, and d_{p} are the characteristic length, the area, the volume, and the diameter of the particle. To assess extra-particle temperature gradients (ΔT_{e}),[12] Eq. 8 was applied,

$$\Delta T_{\text{e}} = \beta_{\text{e}} Ca = \frac{-\Delta H_{\text{r}} k_{\text{f}} c_{\text{b}} Ca}{h T_{\text{b}}} \qquad \text{Eq. 8}$$

where β_e denotes the external Prater number, T_{b} the temperature in the bulk phase (473 K), h the heat transfer coefficient (estimated at a minimum value of 10 J m^{-2} s^{-1} K^{-1}), and $-\Delta H_{\text{r}}$ the reaction enthalpy (99.3 kJ mol^{-1}). Internal mass transfer limitations were evaluated using the Weisz-Prater criterion (Φ),[14] according to Eq. 9,

$$\Phi = \frac{r_{\text{v,obs}} L^2}{D_{\text{eff}} c_{\text{s}}} \left(\frac{n+1}{2}\right) < 1 \qquad \text{Eq. 9}$$

where L is the characteristic length (0.24 mm), c_s is the surface concentration ($c_s \approx c_b$ in the absence of external mass transfer limitations), and the effective diffusion coefficient, which can be derived via Eq. 10,

$$D_{eff} = \frac{\varepsilon}{\tau}\bar{D} = \frac{\varepsilon}{\tau}\left(\frac{1}{D_{C_2H_2,\,HCl}} + \frac{1}{D_k}\right)^{-1} \quad \text{Eq. 10}$$

where τ is the tortuosity factor (estimated at 3), ε is the particle porosity (estimated at 0.2), $D_{C_2H_2,HCl}$ is the molecular diffusion coefficient ($1.96 \cdot 10^{-5}$ m^2 s^{-1}), and is the Knudsen diffusion coefficient, which is calculated for components i (C_2H_2, HCl) in a cylindrical pore according to Eq. 11,

$$D_{K,i} = 97 r_{pore}\sqrt{\frac{T}{M_i}} \quad \text{Eq. 11}$$

where r_{pore} denotes the pore radius, M_i the molecular weight of component i, and T the temperature (473 K). Intra-particle temperature gradients (ΔT_i) can be calculated using Eq. 12,[13]

$$\Delta T_i = \beta_i T_s = \frac{-\Delta H_r D_{eff} c_b}{\lambda_{eff}} \quad \text{Eq. 12}$$

where β_i denotes the internal Prater number and λ_{eff} the effective thermal conductivity (estimated at 0.5 W K^{-1} m^{-1}).

The results for the 0.2-[$(NH_3)_4Pt$]SO_4 catalyst are given in **Table S18**. The Carberry criterion (Eq. 5) is met in all catalytic tests while the external temperature difference is negligible (<0.01 K), indicating the absence of external mass and heat transfer limitations, respectively. Finally, the temperature gradient within the catalyst particle is negligible for each case, indicating the absence of internal heat transfer limitations. In addition, acetylene hydrochlorination tests over the 0.2-[$(NH_3)_4Pt$]SO_4 catalyst performed at variable flow rates and constant F_T/m_{cat} as well as using catalyst extrudates and particles of different sizes at constant F_T/m_{cat} corroborated the absence of extra- and intraparticle mass-transfer limitations (**Figure 8b**), respectively.

1.5. Kinetic modeling

1.5.1. Approach and notation

The surface reactions were modeled according to either an Eley-Rideal (ER) or a Langmuir-Hinshelwood-Hougen-Watson (LHHW) type mechanism, considering that intermediate inhibition may be present. Provided only metal-HCl interactions are experimentally observed, while metal-C_2H_2 ones are not detected,[10] three different models are developed: (i) ER, where only HCl chemisorbs over the metal; (ii) one-site LHHW, where both HCl and C_2H_2 chemisorb over the metal site; and (iii) two-site LHHW, where HCl and C_2H_2 chemisorb over the metal and neighboring carbon sites, respectively. The corresponding rate equations were derived using the following definitions:

p_i partial pressure of compound i

r_n	reaction rate of elementary step *n*
S	probabilistic parameter for adsorption
N_T	total number of active sites over the surface
k_n, k_{-n}	forward and backward rate constants of elementary step *n*
k_n^0	Arrhenius pre-exponential factor for rate constant k_n
θ_i	surface coverage of compound *i*
$K_i = \frac{k_{ads,i}}{k_{des,i}} = \frac{\theta_i}{p_i}$	equilibrium constant for adsorption of compound *i*

In the ER and one-site LHHW models, all reactants and intermediate species chemisorb on metal sites, $*$:

θ^*	fraction of empty Pt sites

In the two-site LHHW model, C_2H_2, C_2H_3Cl, and the C_2H_3 intermediate chemisorb on carbon sites, $^\#$, while HCl and the Cl intermediate chemisorb on metal sites, $*$:

θ^*	fraction of empty Pt sites
$\theta^\#$	fraction of empty sites in the carbon

1.5.2. Derivation of the ER model

In the ER mechanism, gas-phase C_2H_2 is assumed to react with chemisorbed HCl molecules on the metal sites ($*$). This is described by the following elementary steps,

Step I: $HCl + * \underset{k_{-1}}{\overset{k_1}{\rightleftarrows}} HCl^*$ $\quad K_1 = \frac{k_1}{k_{-1}} = \frac{\theta_{HCl}}{p_{HCl}\theta^*}$

Step II: $HCl^* + C_2H_2 \xrightarrow{k_2} \overbrace{(Cl)(C_2H_3)^*}^{I}$ (rds) $\quad r = k_2 s N_T \theta_{HCl} p_{C_2H_2}$

Step III: $(Cl)(C_2H_3)^* \underset{k_{-3}}{\overset{k_3}{\rightleftarrows}} C_2H_3Cl^*$ $\quad K_3 = \frac{k_3}{k_{-3}} = \frac{\theta_{VCM}}{\theta_I}$

Step IV: $C_2H_3Cl^* \underset{k_{-4}}{\overset{k_4}{\rightleftarrows}} C_2H_3Cl + *$ $\quad K_4 = \frac{k_4}{k_{-4}} = \frac{p_{C_2H_3Cl}\theta^*}{\theta_{C_2H_3Cl}}$

where I represents the intermediate species from the reaction between dissociated HCl^* and C_2H_2 (in Steps II and III) over the same metal site. The addition of H to C_2H_2 in Step II is assumed to be the rate-determining step (rds). Site balance over the metal site results in Eq. 13,

$$\theta^* + \theta_{HCl} + \theta_I + \theta_{C_2H_3Cl} = 1$$

$$\theta^*\left(1 + K_1 p_{HCl} + \frac{p_{C_2H_3Cl}}{K_3 K_4} + \frac{p_{C_2H_3Cl}}{K_4}\right) = 1$$

$$\theta^* = \frac{1}{\left(1 + K_1 p_{\mathrm{HCl}} + p_{\mathrm{C_2H_3Cl}}\left(\frac{1}{K_4} + C_0\right)\right)}; \; C_0 = \left(K_3 K_4\right)^{-1} \quad \text{Eq. 13}$$

Finally, the concentration of empty sites is assumed to not change with time under steady-state conditions, based on the pseudo-steady-state hypothesis (PSSH). Combining this assumption with site balance results in the overall rate expression in Eq. 14,

$$\begin{aligned} r_{\mathrm{VCM}} &= k_2 s N_T p_{\mathrm{C_2H_2}} \theta_{\mathrm{HCl}} \\ &= k_2 s N_T K_1 p_{\mathrm{HCl}} p_{\mathrm{C_2H_2}} \theta^* \\ &= \frac{k_2 s N_{\mathrm{T}} K_1 p_{\mathrm{HCl}} p_{\mathrm{C_2H_2}}}{\left(1 + K_1 p_{\mathrm{HCl}} + p_{\mathrm{C_2H_3Cl}}\left(\frac{1}{K_4} + C_0\right)\right)}; \; C_0 = \left(K_3 K_4\right)^{-1} \end{aligned} \quad \text{Eq. 14}$$

1.5.3. Derivation of the one-site LHHW Model

The one-site LHHW model assumes that the reactants compete for the same metal site (*) over the catalytic surface, where only the HCl molecules that bind metal sites with C_2H_2 chemisorbed yield VCM. This is described by the following elementary steps,

Step I: $C_2H_2 + {}^* \underset{k_{-1}}{\overset{k_1}{\rightleftarrows}} C_2H_2^*$ $\quad K_1 = \frac{k_1}{k_{-1}} = \frac{\theta_{\mathrm{C_2H_2}}}{p_{\mathrm{C_2H_2}} \theta^*}$

Step II: $\mathrm{HCl} + {}^* \underset{k_{-2}}{\overset{k_2}{\rightleftarrows}} \mathrm{HCl}^*$ $\quad K_2 = \frac{k_2}{k_{-2}} = \frac{\theta_{\mathrm{HCl}}}{p_{\mathrm{HCl}} \theta^*}$

Step III: $C_2H_2^* + \mathrm{HCl} \underset{k_{-3}}{\overset{k_3}{\rightleftarrows}} \overbrace{(\mathrm{H})(\mathrm{C_2H_2Cl})^*}^{\mathrm{I}}$ $\quad K_3 = \frac{\theta_{\mathrm{I}}}{\theta_{\mathrm{C_2H_2}} p_{\mathrm{HCl}}}$

Step IV: $(\mathrm{H})(\mathrm{C_2H_2Cl})^* \xrightarrow{k_4} \mathrm{C_2H_3Cl}^*$ (rds) $\quad r = k_4 \mathrm{s} N_{\mathrm{T}} \theta_{\mathrm{I}}$

Step V: $\mathrm{C_2H_3Cl}^* \underset{k_{-5}}{\overset{k_5}{\rightleftarrows}} \mathrm{C_2H_3Cl} + {}^*$ $\quad K_5 = \frac{k_5}{k_{-5}} = \frac{p_{\mathrm{C_2H_3Cl}} \theta^*}{\theta_{\mathrm{C_2H_3Cl}}}$

where I represents the $(\mathrm{H})(\mathrm{C_2H_2Cl})^*$ intermediate species from the dissociative addition of HCl to $C_2H_2^*$ in Step III, followed by intra-intermediate addition of (H) to form $(\mathrm{C_2H_2Cl})^*$ in Step IV which is assumed to be the rds. To account for the spatial isolation of metal sites in reality, only the addition of gas-phase HCl to chemisorbed C_2H_2 is possible in Step III. Nonetheless, the current LHHW model accounts for the chemisorption of both reactants, unlike an ER-mechanism where only one of the reactants is chemisorbed. The adsorption of HCl is an important step in the proposed mechanism since it affects the surface coverage and thus, the rate of the reaction. Site balance over the metal site gives Eq. 15,

$$\theta^* + \theta_{\mathrm{C_2H_2}} + \theta_{\mathrm{HCl}} + \theta_{\mathrm{I}} + \theta_{\mathrm{C_2H_3Cl}} = 1$$

$$\theta^*\left(1+K_1p_{C_2H_2}+K_2p_{HCl}+K_1K_3p_{C_2H_2}p_{HCl}+\frac{p_{C_2H_3Cl}}{K_5}\right)=1$$

$$\theta^*=\frac{1}{\left(1+K_1p_{C_2H_2}+K_2p_{HCl}+K_1K_3p_{C_2H_2}p_{HCl}+\frac{p_{C_2H_3Cl}}{K_5}\right)} \quad \text{Eq. 15}$$

The concentration of empty sites is assumed to not change with time under steady-state conditions, based on the PSSH. Combining this assumption with site balance results in the overall rate expression in Eq. 16,

$$\begin{aligned} r_{VCM} &= k_4 \mathrm{s} N_T \theta_I \\ &= k_4 s N_T K_1 K_3 p_{C_2H_2} p_{HCl} \theta^* \\ &= \frac{k_4 s N_T K_1 K_3 p_{C_2H_2} p_{HCl}}{\left(1+K_1p_{C_2H_2}+K_2p_{HCl}+C_1p_{C_2H_2}p_{HCl}+\frac{p_{C_2H_3Cl}}{K_5}\right)};\ C_1=\left(K_1K_3\right)^{-1} \end{aligned} \quad \text{Eq. 16}$$

1.5.4. Derivation of the two-site LHHW model

The two-site LHHW mechanism involves the adsorption of HCl and C_2H_2 on distinct and neighboring metal (*) and carbon ($^\#$) sites, respectively. This is described by the following elementary steps,

Step I: $C_2H_2 + {}^\# \underset{k_{-1}}{\overset{k_1}{\rightleftarrows}} C_2H_2^\#$ $\qquad K_1=\frac{k_1}{k_{-1}}=\frac{\theta_{C_2H_2}}{p_{C_2H_2}\theta^\#}$

Step II: $HCl + {}^* \underset{k_{-2}}{\overset{k_2}{\rightleftarrows}} HCl^*$ $\qquad K_2=\frac{k_2}{k_{-2}}=\frac{\theta_{HCl}}{p_{HCl}\theta^*}$

Step III: $C_2H_2^\# + HCl^* \xrightarrow{k_3} C_2H_3^\# + Cl^*$ (rds) $r=k_3\mathrm{s}N_T\theta_{C_2H_2}\theta_{HCl}$

Step IV: $C_2H_3^\# + Cl^* \xrightarrow{k_4} C_2H_3Cl^\# + {}^*$

Step V: $C_2H_3Cl^\# \underset{k_{-5}}{\overset{k_5}{\rightleftarrows}} C_2H_3Cl + {}^\#$ $\qquad K_5=\frac{k_5}{k_{-5}}=\frac{p_{C_2H_3Cl}\theta^\#}{\theta_{C_2H_3Cl}}$

Applying the PSSH to the $C_2H_3^\#$ and Cl^* intermediates results in Eq. 17,

$$\frac{1}{N_T}\frac{d[C_2H_3^\#]}{dt}=k_3\theta_{C_2H_2}\theta_{HCl}-k_4\theta_{C_2H_3}\theta_{Cl}=0$$

$$\frac{1}{N_T}\frac{d[Cl^*]}{dt}=k_3\theta_{C_2H_2}\theta_{HCl}-k_4\theta_{C_2H_3}\theta_{Cl}=0 \quad \text{Eq. 17}$$

Since the initial coverages of the C_2H_3 and Cl intermediates are equal, their coverages will be equal at any point in time. Computing the fractional site coverage of intermediate(s) gives Eq. 18,

$$k_3\theta_{C_2H_2}\theta_{HCl}-k_4\theta_{C_2H_3}\theta_{Cl}=0$$

$$k_3 K_1 p_{C_2H_2} \theta^{\#} K_2 p_{HCl} \theta^{*} = k_4 \theta_{C_2H_3}^2 = k_4 \theta_{Cl}^2$$

$$\theta_{C_2H_3}^2 = \theta_{Cl}^2 = \left(\frac{k_3 K_1 K_2}{k_4} p_{C_2H_2} p_{HCl} \right) \theta^{*} \theta^{\#}$$

$$\theta_{C_2H_3} = \theta_{Cl} = \left(\frac{k_3 K_1 K_2}{k_4} p_{C_2H_2} p_{HCl} \right)^{1/2} \left(\theta^{*} \theta^{\#} \right)^{1/2} \qquad \text{Eq. 18}$$

The metal site balance results in Eq. 19,

$$\theta^{*} + \theta_{HCl} + \theta_{Cl} = 1$$

$$\theta^{*} + K_2 p_{HCl} \theta^{*} + \left(\frac{k_3 K_1 K_2}{k_4} p_{C_2H_2} p_{HCl} \right)^{1/2} \left(\theta^{*} \theta^{\#} \right)^{1/2} = 1$$

$$\theta^{*} \left(1 + K_2 p_{HCl} + \left(\frac{k_3 K_1 K_2}{k_4} p_{C_2H_2} p_{HCl} \right)^{1/2} \left(\frac{\theta^{\#}}{\theta^{*}} \right)^{1/2} \right) = 1$$

$$\theta^{*} = \frac{1}{\left(1 + K_2 p_{HCl} + \left(\frac{k_3 K_1 K_2}{k_4} p_{C_2H_2} p_{HCl} \right)^{1/2} \left(\frac{\theta^{\#}}{\theta^{*}} \right)^{1/2} \right)} \qquad \text{Eq. 19}$$

The carbon site balance results in Eq. 20,

$$\theta^{\#} + \theta_{C_2H_2} + \theta_{C_2H_3} + \theta_{C_2H_3Cl} = 1$$

$$\theta^{\#} + K_1 p_{C_2H_2} \theta^{\#} + \frac{p_{C_2H_3Cl} \theta^{\#}}{K_5} + \left(\frac{k_3 K_1 K_2}{k_4} p_{C_2H_2} p_{HCl} \right)^{1/2} \left(\theta^{*} \theta^{\#} \right)^{1/2} = 1$$

$$\theta^{\#} \left(1 + K_1 p_{C_2H_2} + \frac{p_{C_2H_3Cl}}{K_5} + \left(\frac{k_3 K_1 K_2}{k_4} p_{C_2H_2} p_{HCl} \right)^{1/2} \left(\frac{\theta^{*}}{\theta^{\#}} \right)^{1/2} \right) = 1$$

$$\theta^{\#} = \frac{1}{\left(1 + K_1 p_{C_2H_2} + \frac{p_{C_2H_3Cl}}{K_5} + \left(\frac{k_3 K_1 K_2}{k_4} p_{C_2H_2} p_{HCl} \right)^{1/2} \left(\frac{\theta^{*}}{\theta^{\#}} \right)^{1/2} \right)} \qquad \text{Eq. 20}$$

The overall rate expression is ultimately calculated according to Eq. 21,

$$r_{VCM} = k_3 s N_T \theta_{C_2H_2} \theta_{HCl}$$

$$= k_3 s N_T K_1 K_2 p_{C_2H_2} p_{HCl} \theta^{*} \theta^{\#}$$

$$= \frac{k_3 s N_T K_1 K_2 p_{C_2H_2} p_{HCl}}{\left(1 + K_2 p_{HCl} + S^{1/2} \left(\frac{\theta^{\#}}{\theta^{*}} \right)^{1/2} \right) \left(1 + K_1 p_{C_2H_2} + \frac{p_{C_2H_3Cl}}{K_5} + S^{1/2} \left(\frac{\theta^{*}}{\theta^{\#}} \right)^{1/2} \right)}; \; S = \left(\frac{k_3 K_1 K_2}{k_4} p_{C_2H_2} p_{HCl} \right) \qquad \text{Eq. 21}$$

The greater complexity of the two-site LHHW model requires a thorough analysis of the region of feasibility, equation constraints, and sensitivity of each modelled parameter in order for numerical

convergence towards the global optimum to be reliably attained. As such, (i) the non-linear system of equations of partial coverage must be solved while ensuring all solutions lie between 0 and 1, and (ii) the non-linear least squares (NLLS), with domain constraints for the model by ensuring a reasonable mean-squared error (MSE) landscape, must be computed to attain the global minimum. The fraction of empty sites covered by HCl and C_2H_2 can be generalized by the modification shown in Eq. 22,

$$\left.\begin{aligned} \theta^* &= \frac{1}{\left(1 + K_2 p_{\mathrm{HCl}} + \left(\frac{k_3 K_1 K_2}{k_4} p_{\mathrm{C_2H_2}} p_{\mathrm{HCl}}\right)^{1/2} \left(\frac{\theta^\#}{\theta^*}\right)^{1/2}\right)} \\ \theta^\# &= \frac{1}{\left(1 + K_1 p_{\mathrm{C_2H_2}} + \frac{p_{\mathrm{C_2H_3Cl}}}{K_5} + \underbrace{\left(\frac{k_3 K_1 K_2}{k_4} p_{\mathrm{C_2H_2}} p_{\mathrm{HCl}}\right)}_{S}^{1/2} \left(\frac{\theta^*}{\theta^\#}\right)^{1/2}\right)} \end{aligned}\right\} \xrightarrow{\text{Generalization}} x = \frac{1}{1 + C + \left(S\frac{y}{x}\right)^{1/2}}$$

Eq. 22

where

$$C = \begin{pmatrix} K_2 p_{\mathrm{HCl}} & \text{in the case of } \theta^* \\ K_1 p_{\mathrm{C_2H_2}} + \frac{p_{\mathrm{C_2H_3Cl}}}{K_5} & \text{in the case of } \theta^\# \end{pmatrix}$$

Converting Eq. 22 to make C the dependent variable results in a function describing isopleths for θ^* and $\theta^\#$, where every curve shows the variation of one of the partial coverages while keeping the other constant, as shown in Eq. 23,

$$C = \frac{1 - \left(x + \sqrt{Sxy}\right)}{x}$$ Eq. 23

Any two curves showing variation of partial coverages intersect at a single point which marks the minimum boundary point, and the region between the curves represents the existence domain of feasible solutions.

1.5.5. Overall rate calculation and parameter fitting

The rate constant appearing in the overall rate expression, k_n, is given by Eq. 24 following the Arrhenius rate law,

$$k_{\mathrm{n}} = k_{\mathrm{n}}^0 \exp\left(-\frac{E_{\mathrm{a}}}{RT}\right)$$ Eq. 24

where the activation energy, E_a, is measured to be 37 kJ mol^{-1} as shown in **Figure S11**.

The catalytic reactor was modeled as a plug flow reactor (PFR) governed by chemical reaction and convection, with no radial mixing or axial dispersion. The change in molar flow rate, dF_i, of each

component i in the gas phase, over an infinitesimal slice of the catalyst bed with mass dm_{cat}, is given by Eq. 25,

$$\frac{dF_i}{dm_{cat}} = v_i r \qquad \text{Eq. 25}$$

where v_i is the stoichiometric coefficient of component i. Ultimately, these equations are solved with least-squares fitting algorithm shown in Eq. 26,

$$Z = \sum_{m=1}^{n} \left[r_{\exp,m} - r_{\text{pred},m} \right]^2 \qquad \text{Eq. 26}$$

where Z is the objective function to be minimized, $r_{\exp,m}$ is the observed reaction rate for the mth experiment, and $r_{\text{pred},m}$ is the predicted reaction rate for the corresponding experimental data point. Multiple iterations were conducted to obtain the values of the model parameters (kinetic constants), which were incorporated back into Eq. 25 to calculate the predicted VCM flow rates.

1.6. Computational methods

DFT simulations were performed using the Vienna Ab initio Simulation Package (VASP).[15,16] The generalized gradient approximation of the Perdew-Burke-Ernzerhof functional was used to obtain the exchange-correlation energies with dispersion contributions introduced via Grimme's DFT-D3 approach.[17,18] Projector augmented wave for the core and plane waves with a cut-off energy of 450 eV for the valence were chosen with spin polarization allowed when needed.[19,20] A vacuum region between slabs of at least 12 Å and a dipole correction along the *z*-axis were employed for slab models.[21] For all investigated systems, structures were relaxed using convergence criteria of 10^{-4} and 10^{-5} eV for the ionic and electronic steps, respectively. To describe the diverse chemical environments in AC, a set of five coordination sites was constructed for the simulation of the AC support, comprising slabs (6×6) of graphitic carbon with O- and S-functionalities (**Figure S8**): (i) tetraketone ($keto_4$), (ii) monothiophene (S), and (ii) triketone-thiophene ($keto_3$-S) coordination sites. The AC-supported Pt single atom catalyst was modeled by placing $PtCl_2$, $Pt(NH_3)_2$, $Pt(OH)_2$, and $Pt(NO_3)_2$ moieties at the center of the distinct AC coordination sites. Physisorbed molecules were considered to preserve ~2/3 and ~1/3 of their gas phase translational and rotational entropy,[22] respectively, as translation over the carbon plane was feasible (i.e., barrier lower than 0.7 eV).

1.7. Evaluation of metal precursor toxicity and reactivity

Information on the toxicity of the platinum precursors was gathered following the Classification, Labelling, and Packaging (CLP) regulation from the European Chemicals Agency (ECHA).[23] On its website,[24] the ECHA reports all necessary information for a given metal precursor, retrieved via the associated CAS number. As the $[(NH_3)_4Pt]SO_4$ and $[(NH_3)_4Pt]$citrate metal precursors were custom-synthesized for this study, neither the CAS number nor information on their toxicity are available. The toxicity of all other six metal precursors was evaluated according to the following methodology. All metal precursors were assessed in their solid form with the exception of $Pt(NO_3)_4$,

assessed in aqueous solution form as no information on its solid form could be retrieved. The ECHA reports and classifies the hazards of a substance by assigning a certain hazard class and associated category code. A total of eleven health and environmental hazard classes were considered. Health hazard classes are acute toxicity, specific target organ toxicity – repeated exposure (STOT-RE), skin irritation, skin corrosion, skin sensitization, respiratory sensitization, eye damage, eye irritation, and reproductive toxicity. Environmental hazard classes are aquatic acute, and aquatic chronic. Hazard category codes qualitatively represent the hazard severity within the class under consideration. In this study, for each hazard class associated with a given platinum precursor, category codes were sorted in a set from least to most severe according to their qualitative description in the CLP regulation.[23] Thereby, the category codes were mapped to a numerical value ranging from one to N, where N corresponds to the highest qualitative toxicity level within the hazard class. This enabled us to assign an overall toxicity score for a given metal precursor, TS_p, by aggregation of all hazard scores through normalization and application of weight factors, by Eq. 27:

$$TS_{p\in P} = \sum_{i\in I} \frac{x_{ip} - \underline{X}_i}{\overline{X}_i - \underline{X}_i} \qquad \forall p \in P \qquad \text{Eq. 27}$$

where

$$\underline{X}_i = \min_{p\in P}\left\{x_{ip}\right\} \qquad \forall i \in I$$

$$\overline{X}_i = \max_{p\in P}\left\{x_{ip}\right\} \qquad \forall i \in I$$

$$I := \left\{\begin{array}{c}\text{Acute toxicity,}\\ \text{STOT RE,}\\ \text{Skin irritation,}\\ \text{Skin corrosion,}\\ \text{Skin sensitivity,}\\ \text{Respiratory sensitivity,}\\ \text{Eye damage,}\\ \text{Eye irritation,}\\ \text{Reproductive toxicity,}\\ \text{Aquatic acute,}\\ \text{Aquatic chronic}\end{array}\right\}$$

$$P := \left\{\begin{array}{c}\mathrm{H_2PtCl_6},\\ \mathrm{K_2PtCl_4},\\ \mathrm{[(NH_3)_4Pt]Cl_2},\\ \mathrm{[(NH_3)_4Pt](HCO_3)_2},\\ \mathrm{Na_2PtOH_6},\\ \mathrm{Pt(NO_3)_4}\end{array}\right\}$$

where x_{ip} is the mapped value for a given hazard class *i,* and precursor *p,* from a set of hazard classes *I* and precursors *P*.

Furthermore, the reactivity of the metal precursors with common chemical compounds in laboratories was assessed. Specifically, 14 chemical compounds were selected as representatives of different categories of hazardous substances.[25] The reactivity evaluation was performed using the Chemical Reactivity Worksheet (CRW4), developed by the National Oceanic and Atmospheric Administration, NOAA, in collaboration with AIChE.[26] This software predicts the compatibility of chemicals that react in pairs, simulating a bicomponent equimolar mixture and predicting the hazard consequences of the mixing. Chemicals compounds are classified according to sixty-eight reactivity groups. While the software database contains this information for over five-thousand common chemical compounds, the classification of metal precursors – some of which were custom-synthesized for this study – into reactivity groups was not available. Hence, a tailored classification was developed in this study, based on the chemical structure and properties (e.g., pH, oxidizing properties). The prediction of reactivity for a specific mixture composed of a metal precursor with a chemical compound is conducted by considering the combination of each of their respective reactive groups. Predictions are based on a database of past incidents of chemical compounds belonging to the same reactivity groups. The resulting predicted hazards are classified according to the following categories: exothermic reaction at ambient temperature; reaction liberated gaseous product and may cause pressurization, reaction may be particularly intense, violent or explosive, reaction product may be corrosive, reaction products may be explosive or sensitive to shock or friction, reaction product may be flammable, reaction products may be toxic, reaction products may be unstable above ambient temperature. If a given reactivity hazard is predicted for a mixture of a metal precursor with a chemical compound, it is assigned a value of one, otherwise, it is assigned a value of zero. This enabled us to assign a global hazard reactivity score for a given metal precursor, RS_p, by aggregation of all reactivity hazard values through normalization and application of weight factors, by Eq. 28:

$$RS_{p\in P} = \sum_{c\in C} \frac{x_{cp} - \underline{X}_c}{\bar{X}_c - \underline{X}_c} \qquad \forall p \in P \qquad \text{Eq. 28}$$

where

$$\underline{X}_c = \min_{p\in P}\left\{x_{cp}\right\} \qquad \forall c \in C$$

$$\bar{X}_c = \max_{p\in P}\left\{x_{cp}\right\} \qquad \forall c \in C$$

$$C := \left\{ \begin{array}{c} NaNO_3, \\ HNO_3, \\ CH_3OH, \\ C_2H_6O, \\ C_3H_6O, \\ Ca(OH)_2, \\ NaOH, \\ H_2SO_4, \\ HCl\ (aq), \\ K_2S, \\ H_2S, \\ Cl_2, \\ H_2O, \\ NaCl \end{array} \right\}$$

$$P := \left\{ \begin{array}{c} H_2PtCl_6, \\ K_2PtCl_4, \\ [(NH_3)_4Pt]Cl_2, \\ [(NH_3)_4Pt](HCO_3)_2, \\ Na_2PtOH_6, \\ Pt(NO_3)_4 \end{array} \right\}$$

where x_{cp} is the total number of hazards for the mixture of a chemical c and precursor p, from a set of common chemicals C and precursors P.

2. Supporting tables

Table S1. Composition of AC and selected Pt SACs.

Catalyst	Elemental composition[a]	Pt content[b] / wt%
AC	$C_{0.9047}H_{0.0071}O_{0.0390}N_{0.0405}S_{0.0047}Cl_{0.0072}$	-
0.2-H_2PtCl_6	$C_{0.8968}H_{0.0063}O_{0.0360}N_{0.0415}S_{0.0057}Cl_{0.0075}$	0.18
0.2-$[(NH_3)_4Pt]SO_4$	$C_{0.8984}H_{0.0068}O_{0.0370}N_{0.0450}S_{0.0049}Cl_{0.0085}$	0.18
0.2-$[(NH_3)_4Pt](HCO_3)_2$	$C_{0.8929}H_{0.0070}O_{0.0386}N_{0.0485}S_{0.0045}Cl_{0.0090}$	0.19
0.8-$[(NH_3)_4Pt]SO_4$	$C_{0.8899}H_{0.0074}O_{0.0415}N_{0.0595}S_{0.0059}Cl_{0.0058}$	0.74

[a]Elemental analysis. [b]ICP-OES.

Table S2. Porous properties of AC and selected Pt SACs.

Catalyst	V_{total}[a] / $cm^3\ g^{-1}$	V_{micro}[b] / $cm^3\ g^{-1}$	S_{BET}[c] / $m^2\ g^{-1}$
AC	0.94	0.41	1658
0.2-H_2PtCl_6	0.83	0.46	1478
0.2-$[(NH_3)_4Pt]SO_4$	0.79	0.46	1396
0.2-H_2PtCl_6-u[d]	0.52	0.29	907
0.2-$[(NH_3)_4Pt]SO_4$-u[d]	0.54	0.33	938

[a]Volume of N_2 adsorbed at $p/p_0 = 0.98$. [b]*t*-plot method. [c]BET method. [d]Catalysts after use in acetylene hydrochlorination for 144 h, increasing temperature by 20 K every 48 h, from 433 to 473 K (**Figure 6a**).

Table S3. Surface composition of selected Pt SACs as determined by XPS.

Catalyst	Pt / at%	C / at%	O / at%	N / at%	S / at%	Cl / at%
0.8-$[(NH_3)_4Pt]SO_4$	0.04	96.28	3.36	0.16	0.10	0.06
0.8-$[(NH_3)_4Pt]SO_4$-48h[a]	0.04	95.81	2.89	0.28	0.02	0.97

[a]Catalysts after use in acetylene hydrochlorination for 48 h at 433 K, as indicated in the sample code.

Table S4. Fitting parameters derived from the Pt 4*f* XPS spectra of selected Pt SACs.

Catalyst	Pt-Cl[a]			$Pt-NH_x$[a]			Pt-O[a]		
	Position / eV	FWHM[b] / eV	Area / %	Position / eV	FWHM[b] / eV	Area / %	Position / eV	FWHM[b] / eV	Area / %
0.8-$[(NH_3)_4Pt]SO_4$	-	-	-	73.3	1.7	78	72.6	2.0	22
0.8-$[(NH_3)_4Pt]SO_4$-48h[c]	73.6	2.3	70	73.4	1.2	8	72.4	2.0	22

[a]Assignment based on reference values.[1-6] [b]FWHM: full width at half maximum. [c]Catalysts after use in acetylene hydrochlorination at 433 K for 48 h.

Table S5. Fitting parameters derived from the Cl 2*p* XPS spectra of selected Pt SACs.

Catalyst	Cl-C[a]			Cl-M[a,b]		
	Position / eV	FWHM[c] / eV	Area / %	Position / eV	FWHM[c] / eV	Area / %
0.8-[$(NH_3)_4$Pt]SO_4	-	-	-	198.5	2.5	100
0.8-[$(NH_3)_4$Pt]SO_4-48h[d]	200.5	1.7	66	198.3	2.0	44

[a]Assignment based on reference values.[3] [b]M = Na, K, Pt; contribution assigned to inorganic Cl. [c]FWHM: full width at half maximum. [d]Catalysts after use in acetylene hydrochlorination at 433 K for 48 h.

Table S6. Fitting parameters derived from the O 1*s* XPS spectra of selected Pt SACs.

Catalyst	O-C[a]			O=C[a]		
	Position / eV	FWHM[b] / eV	Area / %	Position / eV	FWHM[b] / eV	Area / %
0.8-$[(NH_3)_4Pt]SO_4$	533.4	2.9	43	531.6	2.5	57
0.8-$[(NH_3)_4Pt]SO_4$-48h[c]	533.4	2.9	55	531.7	2.4	45

[a]Assignment based on reference values.[3,7] [b]FWHM: full width at half maximum. [c]Catalysts after use in acetylene hydrochlorination at 433 K for 48 h.

Table S7. Fitting parameters derived from the S 2*p* XPS spectra of selected Pt SACs.

Catalyst	SO_3H[a]			SO_2[a]			S[a]		
	Position / eV	FWHM[b] / eV	Area / %	Position / eV	FWHM[b] / eV	Area / %	Position / eV	FWHM[b] / eV	Area / %
0.8-$[(NH_3)_4Pt]SO_4$	168.9	1.8	41	167.8	1.8	23	164.1	1.7	36
0.8-$[(NH_3)_4Pt]SO_4$-48h[c]	-	-	-	-	-	-	164.1	2.0	100

[a]Assignment based on reference values.[8] [b]FWHM: full width at half maximum. [c]Catalysts after use in acetylene hydrochlorination at 433 K for 48 h.

Table S8. Fitting parameters derived from the Pt L_3 edge EXAFS spectra of selected Pt SACs.

Catalyst	Coordination	CN[a] / -	σ^{2b} / Å^2	R^c / Å	R_f^d / -
0.2-$[(NH_3)_4Pt]SO_4$	Pt-O/C/N	3.8 ± 0.5	0.005 ± 0.002	2.05 ± 0.02	0.010
	Pt-Cl/S	0.7 ± 0.3	0.002 ± 0.002	2.31 ± 0.02	
0.2-$[(NH_3)_4Pt](HCO_3)_2$	Pt-O/C/N	3.7 ± 0.5	0.005 ± 0.002	2.05 ± 0.02	0.008
	Pt-Cl/S	0.6 ± 0.3	0.002 ± 0.002	2.31 ± 0.02	
0.2-$[(NH_3)_4Pt]Cl_2$	Pt-O/C/N	3.5 ± 0.5	0.005 ± 0.002	2.05 ± 0.02	0.006
	Pt-Cl/S	1.3 ± 0.3	0.002 ± 0.002	2.31 ± 0.02	
0.2-H_2PtCl_6	Pt-O/C/N	0.4 ± 0.5	0.006 ± 0.002	2.06 ± 0.02	0.003
	Pt-Cl/S	3.9 ± 0.4	0.004 ± 0.002	2.34 ± 0.02	

[a]Coordination number. [b]Debye-Waller factor. [c]Coordination shell distance. [d]*R*-factor.

Table S9. Initial activity of Pt SACs (t = h), expressed as initial VCM yield, $Y_{VCM,0}$, and initial turnover frequency, TOF_0, in acetylene hydrochlorination at 433 and 473 K.

Catalyst[a]	$Y_{VCM,0}$ / %		TOF_0 / $mol_{VCM}\ s^{-1}\ mol_{Pt}^{-1}$	
	433 K	473 K	433 K	473 K
0.2-$[(NH_3)_4Pt]SO_4$	22	27	0.35	0.44
0.2-$[(NH_3)_4Pt](HCO_3)_2$	21	26	0.34	0.42
0.2-$[(NH_3)_4Pt]Cl_2$	22	27	0.35	0.44
0.2-$[(NH_3)_4Pt]$citrate	17	-	0.27	-
0.2-K_2PtCl_4	18	-	0.29	-
0.2-$Na_2Pt(OH)_6$	16	-	0.26	-
0.2-$Pt(NO_3)_4$	11	-	0.18	-
0.2-H_2PtCl_6	13	12	0.21	0.20

[a]Reaction conditions: $GHSV(C_2H_2) = 650\ h^{-1}$, $P = 1$ bar, $T = 433$ or 473 K, C_2H_2:HCl:Ar = 40:44:16.

Table S10. Amount of carbonaceous deposits formed over 0.2-$[(NH_3)_4Pt]SO_4$ after use in acetylene hydrochlorination at 433 and 473 K for 48 h.

T / K	Coke content / wt%
433	2.0
473	2.8

Table S11. Fitting parameters derived from the Pt L_3 edge EXAFS spectra of selected Pt SACs under reaction conditions at 433 K.

Catalyst	Coordination	CN[a] / -	σ^{2b} / Å^2	R^c / Å	R_f^d / -
0.8-[$(NH_3)_4Pt$]SO_4-He[e]	Pt-O/C/N	3.9 ± 0.2	0.006 ± 0.002	2.06 ± 0.02	0.010
	Pt-Cl/S	0.4 ± 0.2	0.004 ± 0.002	2.29 ± 0.02	
0.8-[$(NH_3)_4Pt$]SO_4-90[e]	Pt-O/C/N	1.4 ± 0.2	0.006 ± 0.002	2.06 ± 0.02	0.004
	Pt-Cl/S	3.4 ± 0.2	0.004 ± 0.002	2.32 ± 0.02	
0.8-[$(NH_3)_4Pt$]$(HCO_3)_2$-He[e]	Pt-O/C/N	3.8 ± 0.2	0.006 ± 0.002	2.05 ± 0.02	0.019
	Pt-Cl/S	0.4 ± 0.2	0.004 ± 0.002	2.29 ± 0.02	
0.8-[$(NH_3)_4Pt$]$(HCO_3)_2$-90[e]	Pt-O/C/N	2.0 ± 0.2	0.006 ± 0.002	2.06 ± 0.02	0.007
	Pt-Cl/S	3.1 ± 0.2	0.004 ± 0.002	2.34 ± 0.02	
0.8-H_2PtCl_6-He[e]	Pt-O/C/N	0.7 ± 0.2	0.006 ± 0.002	2.06 ± 0.02	0.003
	Pt-Cl/S	3.6 ± 0.2	0.004 ± 0.002	2.34 ± 0.02	
0.8-H_2PtCl_6-90[e]	Pt-O/C/N	0.8 ± 0.2	0.006 ± 0.002	2.05 ± 0.02	0.002
	Pt-Cl/S	3.8 ± 0.2	0.004 ± 0.002	2.34 ± 0.02	

[a]Coordination number. [b]Debye-Waller factor. [c]Coordination shell distance. [d]*R*-factor. [e]Sample code: catalyst under He, 0.8-[$(NH_3)_4Pt$]SO_4-He, and catalyst under reaction conditions for 90 min, 0.8-[$(NH_3)_4Pt$]SO_4-90.

Table S12. Fitting parameters derived from the Pt L_3 edge EXAFS spectra of 0.8-[$(NH_3)_4$Pt]SO_4 under reaction conditions at 433 K.

Catalyst	Coordination	CN^a / -	σ^{2b} / Å^2	R^c / Å	R_f^d / -
0.8-[$(NH_3)_4$Pt]SO_4-He[e]	Pt-O/C/N	4.1 ± 0.2	0.006 ± 0.002	2.04 ± 0.02	0.006
	Pt-Cl/S	0.2 ± 0.2	0.004 ± 0.002	2.29 ± 0.02	
0.8-[$(NH_3)_4$Pt]SO_4-15[e]	Pt-O/C/N	3.8 ± 0.2	0.006 ± 0.002	2.06 ± 0.02	0.003
	Pt-Cl/S	2.6 ± 0.2	0.004 ± 0.002	2.31 ± 0.02	
0.8-[$(NH_3)_4$Pt]SO_4-30[e]	Pt-O/C/N	2.4 ± 0.2	0.006 ± 0.002	2.05 ± 0.02	0.006
	Pt-Cl/S	3.1 ± 0.2	0.004 ± 0.002	2.33 ± 0.02	
0.8-[$(NH_3)_4$Pt]SO_4-45[e]	Pt-O/C/N	2.3 ± 0.2	0.006 ± 0.002	2.06 ± 0.02	0.002
	Pt-Cl/S	3.1 ± 0.2	0.004 ± 0.002	2.33 ± 0.02	
0.8-[$(NH_3)_4$Pt]SO_4-60[e]	Pt-O/C/N	1.6 ± 0.2	0.006 ± 0.002	2.06 ± 0.02	0.016
	Pt-Cl/S	3.7 ± 0.2	0.004 ± 0.002	2.33 ± 0.02	
0.8-[$(NH_3)_4$Pt]SO_4-75[e]	Pt-O/C/N	1.6 ± 0.2	0.006 ± 0.002	2.06 ± 0.02	0.003
	Pt-Cl/S	3.3 ± 0.2	0.004 ± 0.002	2.33 ± 0.02	
0.8-[$(NH_3)_4$Pt]SO_4-90[e]	Pt-O/C/N	1.7 ± 0.2	0.006 ± 0.002	2.05 ± 0.02	0.003
	Pt-Cl/S	3.1 ± 0.2	0.004 ± 0.002	2.32 ± 0.02	

[a]Coordination number. [b]Debye-Waller factor. [c]Coordination shell distance. [d]*R*-factor. [e]Sample code: catalyst under He, 0.8-[$(NH_3)_4$Pt]SO_4-He, and catalyst under reaction conditions, 0.8-[$(NH_3)_4$Pt]SO_4-*Y*. *Y* = time on stream in min.

Table S13. Fitting parameters derived from the Pt L_3 edge EXAFS spectra of 0.8-[$(NH_3)_4Pt$]SO_4 under reaction conditions at 473 K.

Catalyst	Coordination	CN^a / -	σ^{2b} / Å^2	R^c / Å	R_f^d / -
0.8-[$(NH_3)_4Pt$]SO_4-He^e	Pt-O/C/N	3.1 ± 0.2	0.006 ± 0.002	2.04 ± 0.02	0.002
	Pt-Cl/S	1.2 ± 0.2	0.004 ± 0.002	2.32 ± 0.02	
0.8-[$(NH_3)_4Pt$]SO_4-15^e	Pt-O/C/N	0.9 ± 0.2	0.006 ± 0.002	2.05 ± 0.02	0.006
	Pt-Cl/S	2.9 ± 0.2	0.004 ± 0.002	2.33 ± 0.02	
0.8-[$(NH_3)_4Pt$]SO_4-30^e	Pt-O/C/N	0.6 ± 0.2	0.006 ± 0.002	2.06 ± 0.02	0.006
	Pt-Cl/S	3.0 ± 0.2	0.004 ± 0.002	2.32 ± 0.02	
0.8-[$(NH_3)_4Pt$]SO_4-45^e	Pt-O/C/N	0.7 ± 0.2	0.006 ± 0.002	2.04 ± 0.02	0.009
	Pt-Cl/S	2.8 ± 0.2	0.004 ± 0.002	2.33 ± 0.02	
0.8-[$(NH_3)_4Pt$]SO_4-60^e	Pt-O/C/N	0.7 ± 0.2	0.006 ± 0.002	2.06 ± 0.02	0.004
	Pt-Cl/S	3.1 ± 0.2	0.004 ± 0.002	2.33 ± 0.02	
0.8-[$(NH_3)_4Pt$]SO_4-75^e	Pt-O/C/N	0.8 ± 0.2	0.006 ± 0.002	2.06 ± 0.02	0.013
	Pt-Cl/S	2.8 ± 0.2	0.004 ± 0.002	2.33 ± 0.02	

^{a}Coordination number. ^{b}Debye-Waller factor. ^{c}Coordination shell distance. ^{d}*R*-factor. ^{e}Sample code: catalyst under He, 0.8-[$(NH_3)_4Pt$]SO_4-He, and catalyst under reaction conditions, 0.8-[$(NH_3)_4Pt$]SO_4-*Y*. *Y* = time on stream in min.

Table S14. Fitting parameters derived from the Pt L_3 edge EXAFS spectra of 0.8-H_2PtCl_6 under reaction conditions at 433 K.

Catalyst	Coordination	CN[a] / -	σ^{2b} / Å^2	R^c / Å	R_f^d / -
0.8-H_2PtCl_6-He[e]	Pt-O/C/N	0.7 ± 0.2	0.006 ± 0.002	2.04 ± 0.02	0.003
	Pt-Cl/S	3.1 ± 0.2	0.004 ± 0.002	2.34 ± 0.02	
0.8-H_2PtCl_6-15[e]	Pt-O/C/N	0.7 ± 0.2	0.006 ± 0.002	2.04 ± 0.02	0.002
	Pt-Cl/S	3.2 ± 0.2	0.004 ± 0.002	2.34 ± 0.02	
0.8-H_2PtCl_6-30[e]	Pt-O/C/N	0.6 ± 0.2	0.006 ± 0.002	2.02 ± 0.02	0.003
	Pt-Cl/S	3.2 ± 0.2	0.004 ± 0.002	2.34 ± 0.02	

[a]Coordination number. [b]Debye-Waller factor. [c]Coordination shell distance. [d]*R*-factor. [e]Sample code: catalyst under He, 0.8-H_2PtCl_6-He, and catalyst under reaction conditions, 0.8-H_2PtCl_6-*Y*. *Y* = time on stream in min.

Table S15. Fitting parameters derived from the Pt L_3 edge EXAFS spectra of 0.8-H_2PtCl_6 under reaction conditions at 473 K.

Catalyst	Coordination	CN[a] / -	σ^{2b} / Å^2	R^c / Å	R_f^d / -
0.8-H_2PtCl_6-He[e]	Pt-O/C/N	0.3 ± 0.2	0.006 ± 0.002	2.02 ± 0.02	0.005
	Pt-Cl/S	2.8 ± 0.2	0.004 ± 0.002	2.33 ± 0.02	
0.8-H_2PtCl_6-15[e]	Pt-Cl/S	3.1 ± 0.2	0.004 ± 0.002	2.33 ± 0.02	0.011
0.8-H_2PtCl_6-30[e]	Pt-Cl/S	3.2 ± 0.2	0.004 ± 0.002	2.33 ± 0.02	0.011

[a]Coordination number. [b]Debye-Waller factor. [c]Coordination shell distance. [d]*R*-factor. [e]Sample code: catalyst under He, 0.8-H_2PtCl_6-He, and catalyst under reaction conditions, 0.8-H_2PtCl_6-*Y*. *Y* = time on stream in min.

Table S16. Gibbs energy ($\Delta G_{HCl,ads}$, in eV) of hydrogen chloride adsorption on different metal species stabilized on distinct coordination sites in AC, at 433 and 473 K.

Metal species[a]	Coordination site[b]					
	$keto_4$	S	$keto_3$-S	$keto_4$	S	$keto_3$-S
	433 K			473 K		
$Pt(NH_3)_2$	0.25	−0.12	−0.06	0.33	0.04	0.02
$PtCl_2$	0.92	0.39	1.40	0.99	0.46	1.48
$Pt(NO_3)_2$	0.51	0.33	0.46	0.59	0.41	0.54
$Pt(OH)_2$	0.49	0.50	0.44	0.57	0.58	0.52

[a] $\Delta G_{HCl,ads} = G(HCl^*) - G(HCl, gp) - G(PtL_2)$, where $G(HCl^*)$ is the Gibbs free energy of HCl adsorbed on PtL_2, G(HCl, gp) is the Gibbs free energy of the isolated HCl molecule in the gas-phase, and $G(PtL_2)$ is the Gibbs free energy of the PtL_2 system, where $L = NH_3$, Cl^-, NO_3^-, OH^-. [b] The schematic representation of the distinct coordination sites is shown in **Figure S8**.

Table S17. Difference in the Gibbs free energy ($\Delta G_{HCl-C_2H_2,ads}$, in eV) of hydrogen chloride and acetylene adsorption on different metal species stabilized on distinct coordination sites in AC, at 473 K.

Metal species[a]	Coordination site[b]		
	keto$_4$	S	keto$_3$-S
$Pt(NH_3)_2$	−0.49	-[c]	−0.56
$PtCl_2$	−1.65	-[c]	-[c]
$Pt(NO_3)_2$	−0.18	0.01	−1.40
$Pt(OH)_2$	−0.56	−0.36	0.04

[a]$\Delta G_{HCl-C_2H_2,ads} = \Delta G_{HCl,ads} - \Delta G_{C_2H_2,ads}$, where $\Delta G_{HCl,ads}$ is the hydrogen chloride adsorption Gibbs energy and $\Delta G_{C_2H_2,ads}$ is the acetylene adsorption Gibbs free energy. [b]The schematic representation of the distinct coordination sites is shown in **Figure S8**. [c]Formation of volatile metal species.

Table S18. Gibbs energy ($\Delta G_{C_2H_2,ads}$, in eV) of acetylene adsorption on metal-neighboring distinct coordination sites in AC, at 433 and 473 K.

Metal species[a]	Coordination site[b]					
	$keto_4$	S	$keto_3$-S	$keto_4$	S	$keto_3$-S
	433 K			473 K		
$Pt(NH_3)_2$	1.06	-[c]	1.80	1.10	-[c]	1.84
$PtCl_2$	−0.58	-[c]	−0.44	−0.55	-[c]	−0.40
$Pt(NO_3)_2$	0.15	-[c]	−0.63	0.19	-[c]	−0.59
$Pt(OH)_2$	0.07	-[c]	−1.02	0.10	-[c]	−0.94

[a] $\Delta G_{C_2H_2,ads} = G(C_2H_2^*) - G(C_2H_2, gp) - G(PtL_2)$, where $G(C_2H_2^*)$ is the Gibbs free energy of C_2H_2 adsorbed on PtL_2, $G(C_2H_2, gp)$ is the Gibbs free energy of the isolated C_2H_2 molecule in the gas-phase, and $G(PtL_2)$ is the Gibbs free energy of the PtL_2 system, where $L = NH_3$, Cl^-, NO_3^-, OH^-. [b] The schematic representation of the distinct coordination sites is shown in **Figure S8**. [c] Formation of unstable 4-membered ring species.

Table S19. Results for mass and heat transfer limitation criteria.

Catalyst	$r_{v,obs}$ / $mol_{VCM}\ s^{-1}\ m_{cat}^{-3}$	r_{pore}[a] / nm	Ca[b] / -	ΔT_e[c] / K	Φ[d] / -	ΔT_i[e] / K
0.2-$[(NH_3)_4Pt]SO_4$	1.4	2.5	0.0019	0.007	0.19	0.11

[a]Pore radius, estimated using N_2 sorption. [b]Carberry criterion. [c]Extra-particle temperature gradient. [d]Weisz-Prater criterion. [e]Intra-particle temperature gradient.

Table S20. Fitted model parameters for the ER, one-site LHHW, and two-site LHHW mechanisms over 0.2-$[(NH_3)_4Pt]SO_4$, equilibrated for 48 h at 473 K under acetylene hydrochlorination conditions.

Mechanism	Parameter	Value	Unit
ER	K_1	0.37	bar^{-1}
	k_2^0	1.69	s^{-1}
	K_0	0.46	bar
	C_0	302	bar^{-1}
One-site LHHW	K_1	1.39	bar^{-1}
	K_2	11.96	bar^{-1}
	k_4^0	1.28	s^{-1}
	K_5	1.94	bar
	C_1	0.4	bar^{-2}
Two-site LHHW	K_1	1.02	bar^{-1}
	K_2	27.65	bar^{-1}
	k_3^0	0.03	s^{-1}
	k_4	11.66	$mol_{VCM}\ s^{-1}\ g_{cat}$
	K_5	15.51	bar

3. Supporting figures

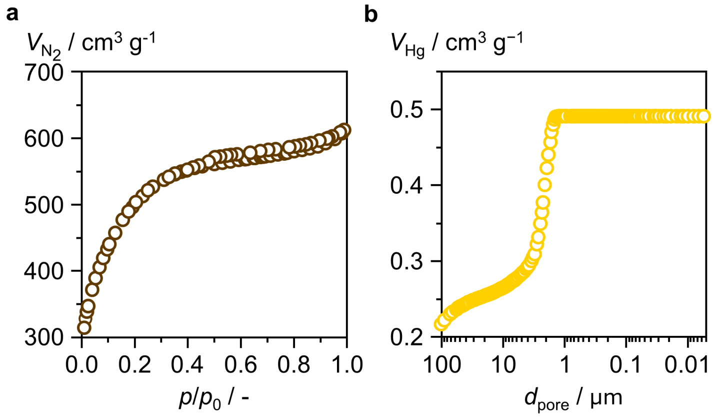

Figure S1. a, Nitrogen sorption isotherm at 77 K and **b,** cumulative intruded volume of mercury at 295 K of AC extrudates.

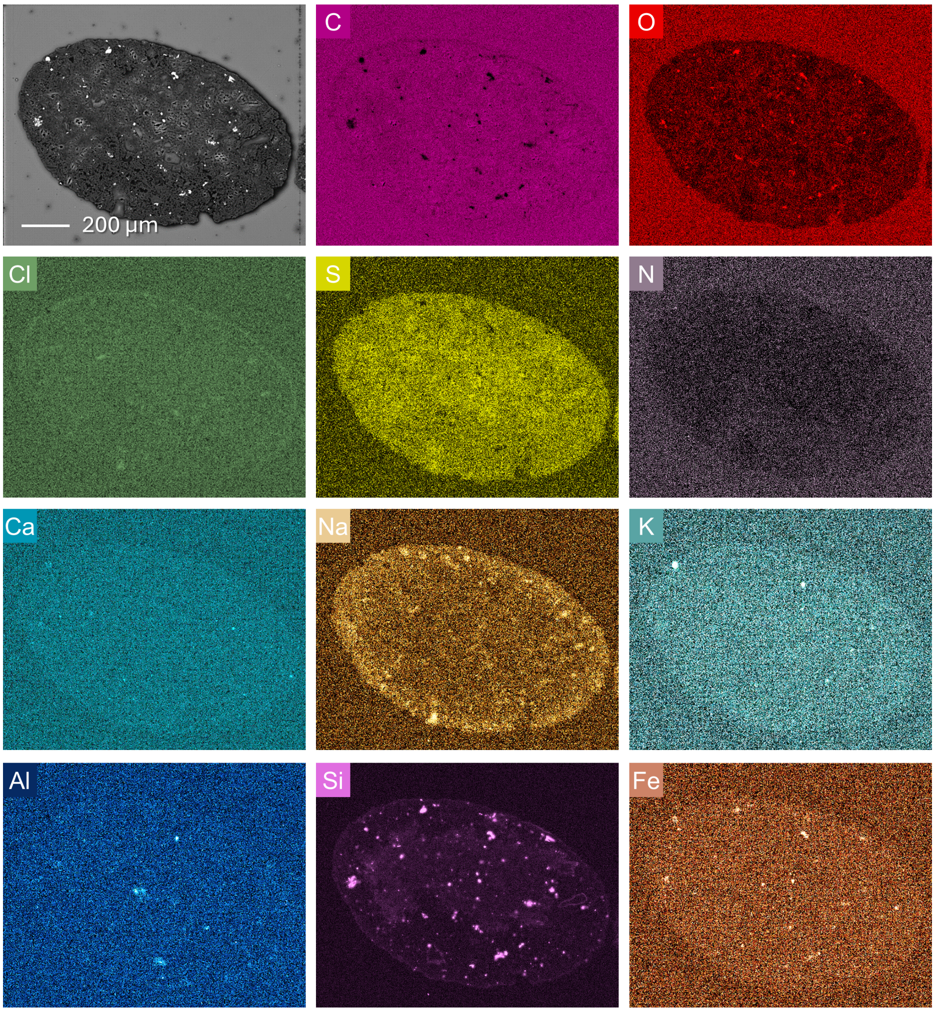

Figure S2. BSE-SEM image and EDXS maps across a transverse-section of an AC extrudate.

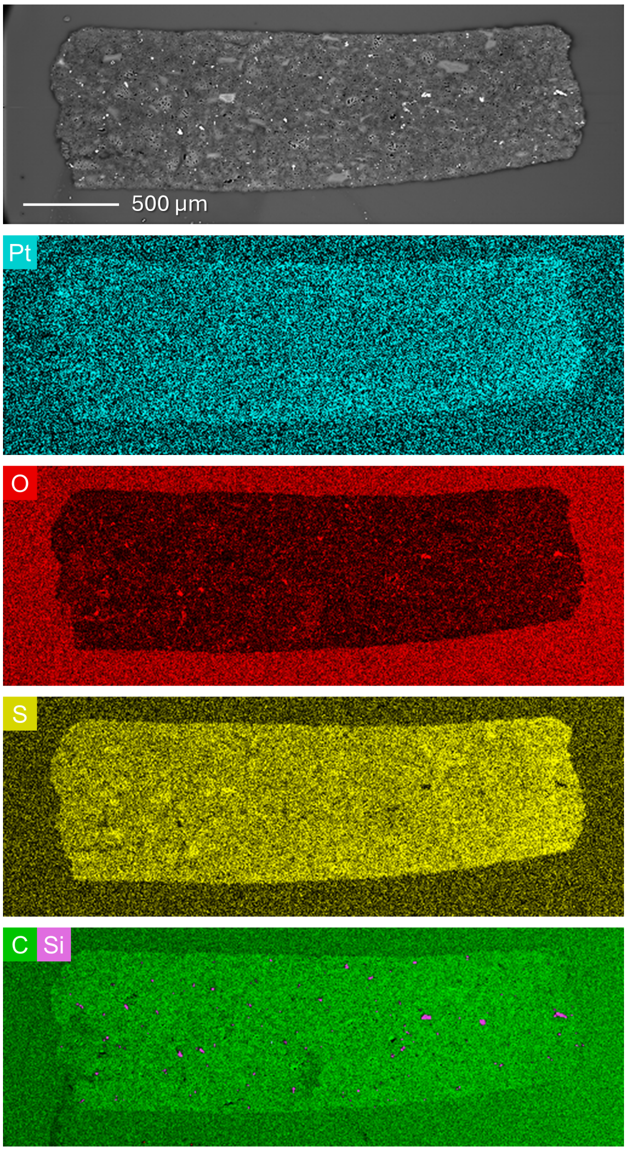

Figure S3. BSE-SEM image and EDXS maps of the most abundant elements across the longitudinal section of a 0.8-$[(NH_3)_4Pt]SO_4$ extrudate.

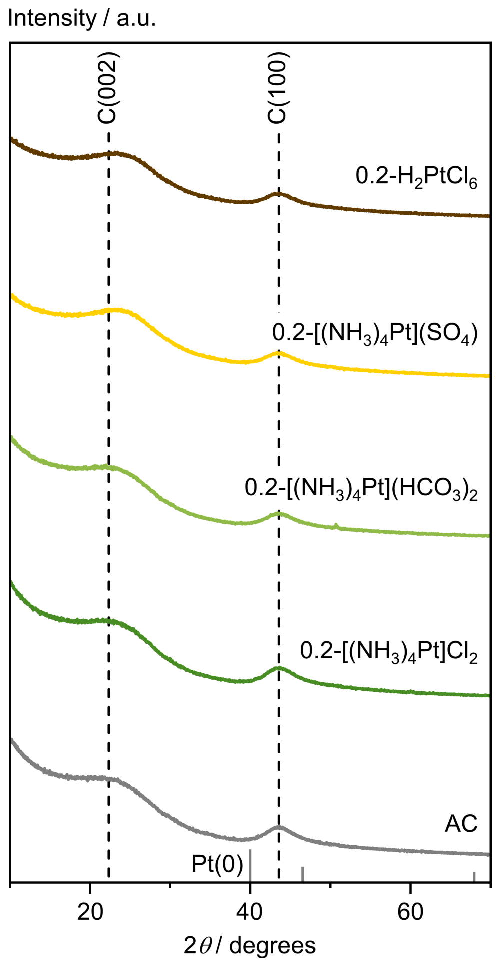

Figure S4. XRD patterns of AC and selected 0.2 wt% Pt SACs. Diffraction peaks of metallic platinum and carbon are indicated by vertical grey bars and dotted black lines, respectively.

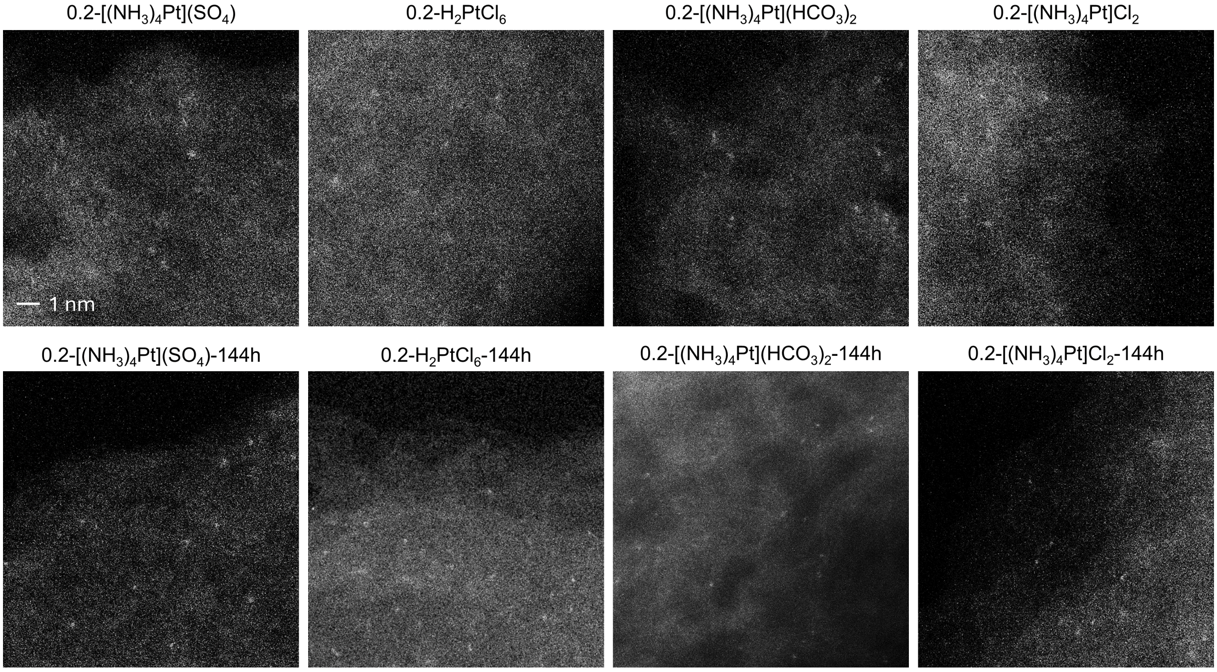

Figure S5. HAADF-STEM images of selected 0.2 wt% Pt SACs, as-prepared and after use in acetylene hydrochlorination for 144 h, increasing temperature by 20 K every 48 h, from 433 to 473 K (**Figure 6a**), denoted as "-144h". Scale bar applies to all images.

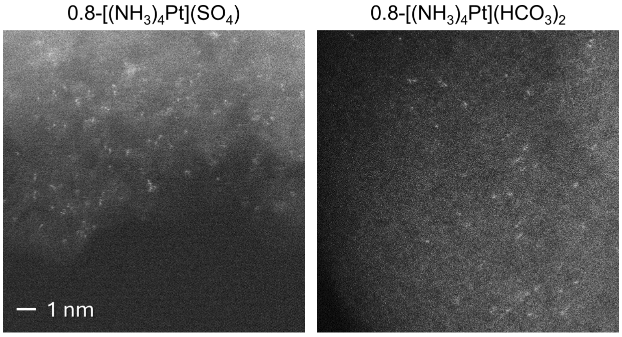

Figure S6. HAADF-STEM images of selected 0.8 wt% Pt SACs. Scale bar applies to all images.

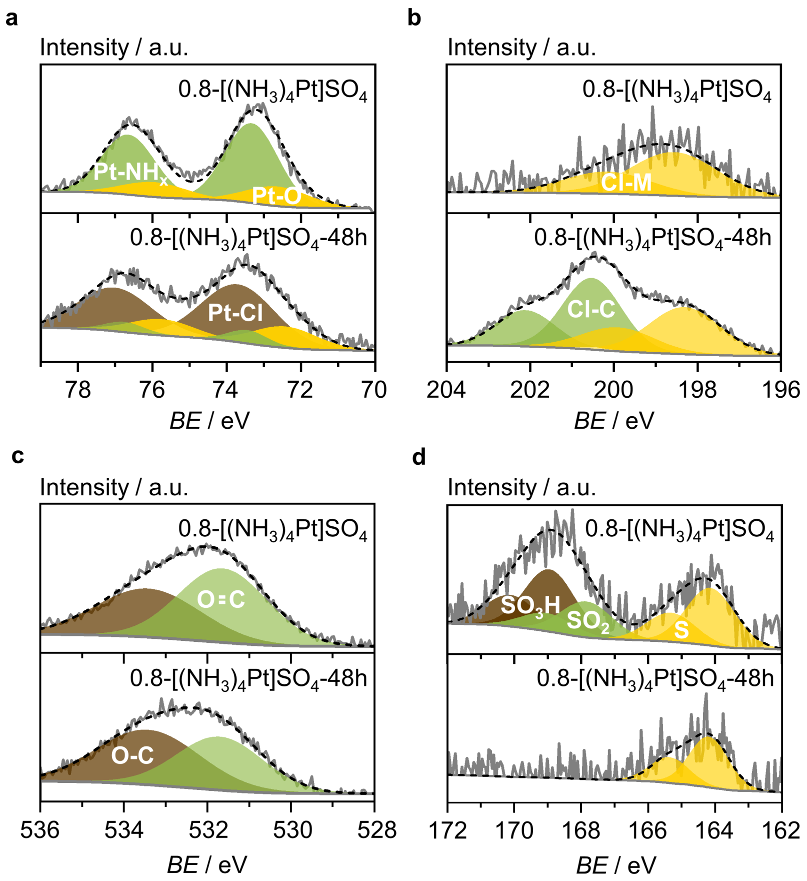

Figure S7. a, Pt 4*f*, **b,** Cl 2*p*, **c,** O 1*s*, and **d,** S 2*p* experimental and fitted XPS spectra of 0.8-[$(NH_3)_4$Pt]SO_4, as-prepared and after use in acetylene hydrochlorination for 48 h at 433 K, respectively denoted as "-48h".

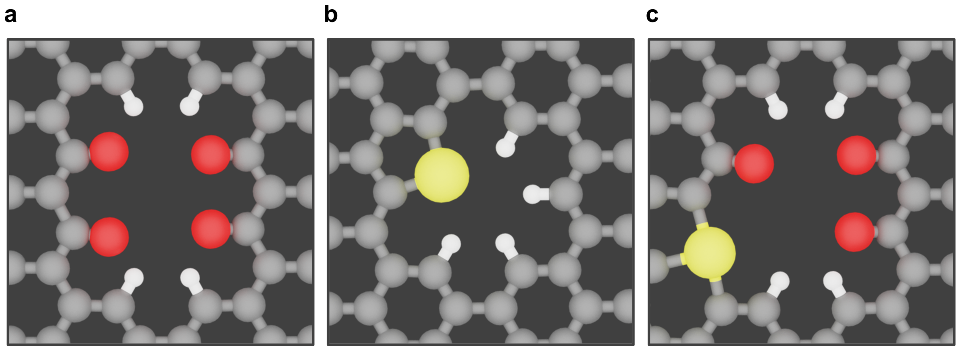

Figure S8. Representation of the coordination sites in AC investigated by DFT simulations. **a,** $keto_4$. **b,** S. **c,** $keto_3$-S.

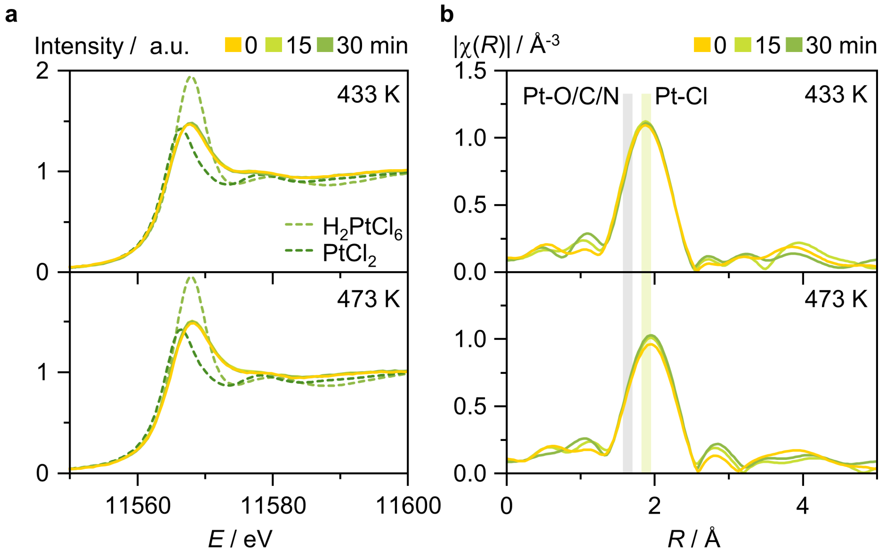

Figure S9. *Operando* Pt L_3 edge **a,** XANES, together with *ex situ* spectra of reference compounds in dotted lines, and **b,** EXAFS of 0.8-H_2PtCl_6.

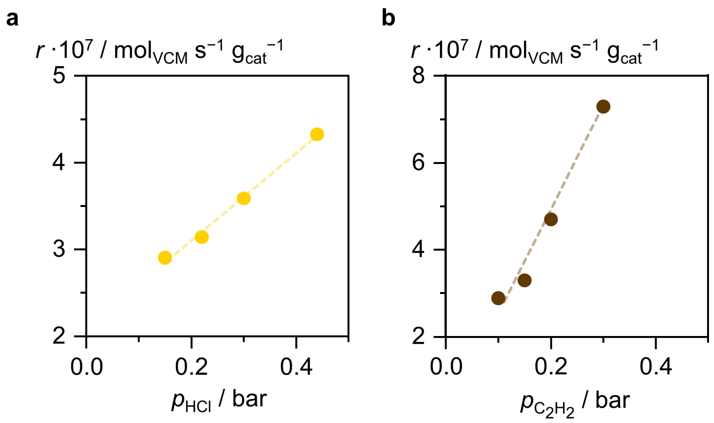

Figure S10. Reaction rate, *r*, over 0.2-$[(NH_3)_4Pt]SO_4$, equilibrated in acetylene hydrochlorination at 473 K for 48 h, as a function of the partial pressure of **a,** HCl and **b,** C_2H_2. Reaction conditions: $T = 453$ K, $m_{cat} = 0.33$ g, $F_T = 10$ cm^3 min^{-1}, $p_{C_2H_2} = 0.1$ bar in **a**, $p_{HCl} = 0.11$ bar in **b**.

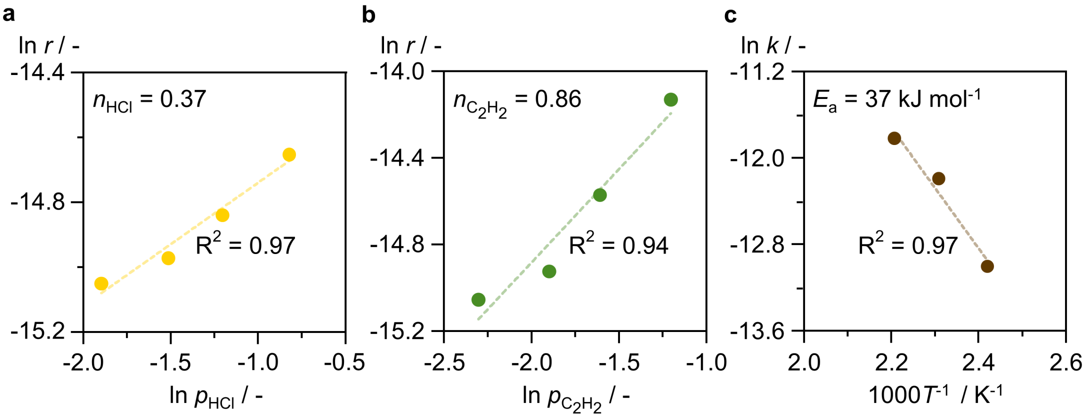

Figure S11. Reaction rate over 0.2-[$(NH_3)_4$Pt]SO_4, equilibrated in acetylene hydrochlorination for 48 h, as a function of the partial pressure of **a,** HCl and **b,** C_2H_2; The partial reaction orders of HCl, n_{HCl}, and C_2H_2, $n_{C_2H_2}$, correspond to the slope of the fitting lines in **a** and **b**, respectively. Reaction conditions: $T = 453$ K, $m_{cat} = 0.33$ g, $F_T = 10$ cm^3 min^{-1}, $p_{C_2H_2} = 0.1$ bar in **a**, $p_{HCl} = 0.11$ bar in **b**. **c,** Arrhenius plot and the corresponding apparent activation energy, E_a. Reaction conditions: $m_{cat} = 0.33$ g, $F_T = 10$ cm^3 min^{-1}, $p_{C_2H_2} = 0.1$ bar, $p_{HCl} = 0.11$ bar.

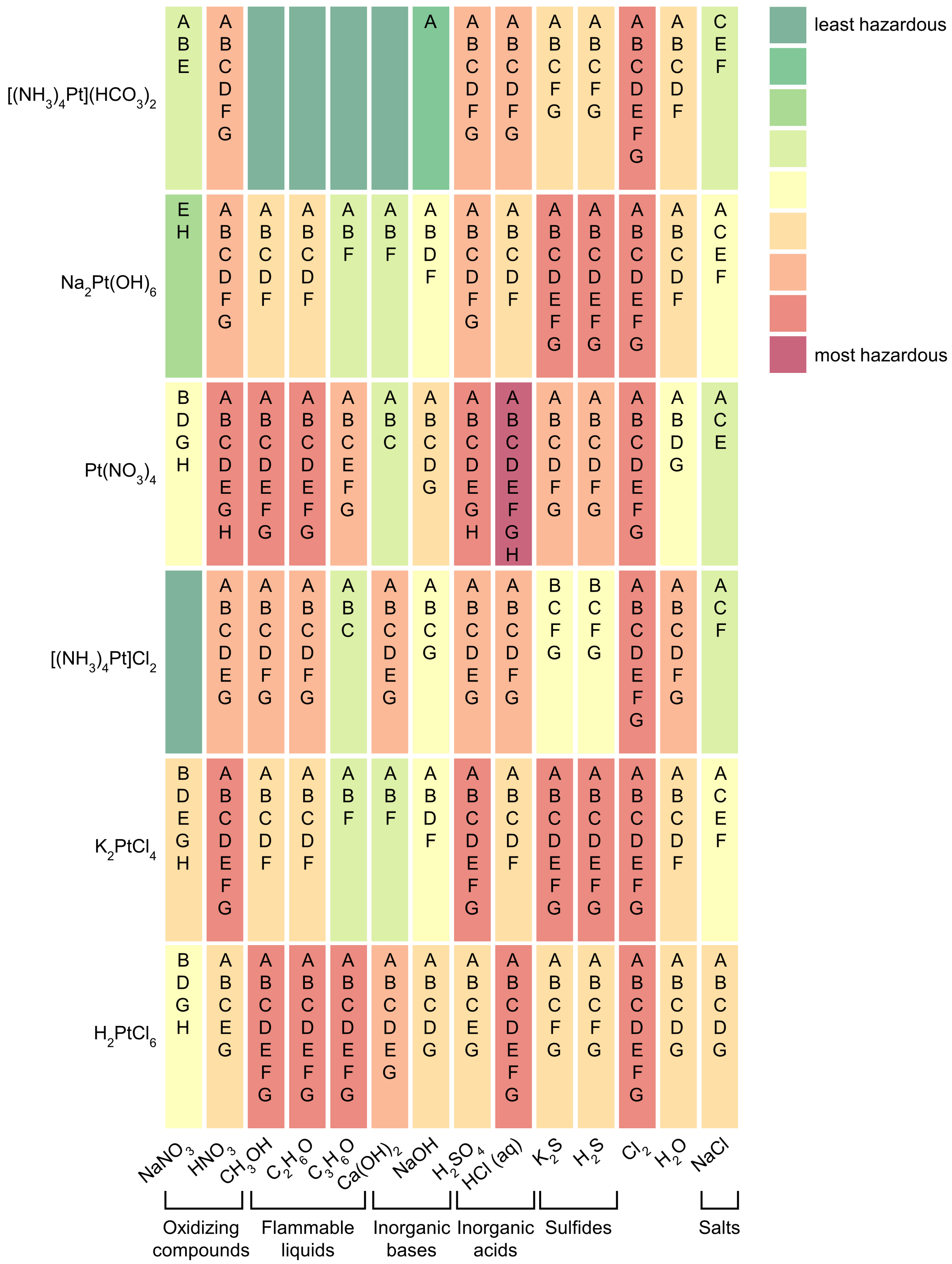

Figure S12. Assessment of metal precursor reactivity with common chemical compounds, selected as representatives of classes of different hazardous substances. Predicted hazards: A, exothermic reaction at ambient temperature; B, Reaction liberated gaseous products and may cause pressurization; C, reaction may be particularly intense, violent or explosive; D, Reaction products may be corrosive; E, Reaction products may be explosive or sensitive to shock or friction; F, Reaction products may be

flammable; G, Reaction products may be toxic; H, Reaction products may be unstable above ambient temperature**.**

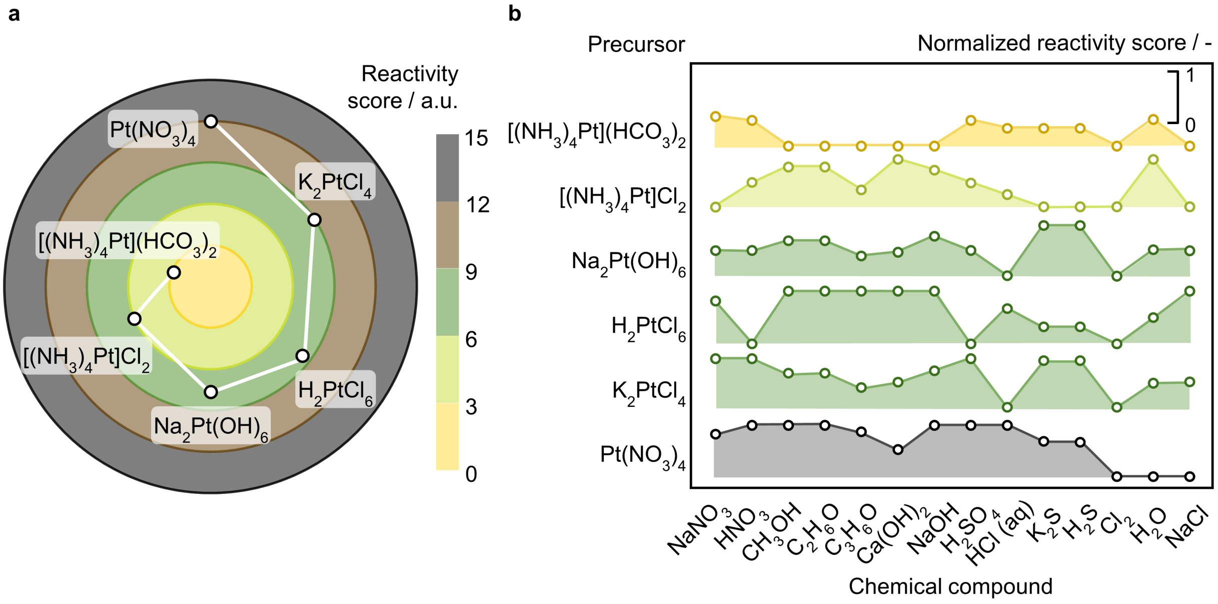

Figure S13. a, Reactivity score of metal precursors, together with **b,** the individual contribution of the reactivity hazard of the metal precursor mixtures with common chemical compounds in laboratories, wherein a normalized reactivity score of zero indicates that the Pt precursor attains the minimum (best) score for a given chemical compound across all Pt precursors, while one corresponds to the maximum (worst) score.

4. Supporting references

1. Kaiser, S.K.; Fako, E.; Manzocchi, G.; Krumeich, F.; Hauert, R.; Clark, A.H.; Safonova, O.V.; López, N.; Pérez-Ramírez, J. Nanostructuring Unlocks High Performance of Platinum Single-Atom Catalysts for Stable Vinyl Chloride Production. *Nat. Catal.* **2020**, *3*, 376. 10.1038/s41929-020-0431-3.
2. Ding, K.; Gulec, A.; Johnson, A.M.; Schweitzer, N.M.; Stucky, G.D.; Marks, L.D.; Stair, P.C. Identification of Active Sites in CO Oxidation and Water-Gas Shift Over Supported Pt Catalysts. *Science* **2015**, *350*, 189. 10.1126/science.aac6368.
3. Moulder, J.F.; Stickle, W.F.; Sobol, P.E.; Bomben, K.D. Handbook of X-ray Photoelectron Spectroscopy. *Perkin-Elmer Corporation* **1995**, United States of America.
4. Maier, F.; Gottfried, J.M.; Rossa, J.; Gerhard, D.; Schulz, P.S.; Schwieger, W.; Wasserscheid, P.; Steinrück, H.P. Surface Enrichment and Depletion Effects of Ions Dissolved in an Ionic Liquid: an X-ray Photoelectron Spectroscopy Study. *Angew. Chem. Int. Ed.* **2006**, *45*, 7778. 10.1002/anie.200602756.
5. Bastl, Z.; Kubelková, L.; Nováková, J. XPS Study of Pt Ammine Decomposition in K Faujasites: Comparison with Bulk Behavior. *Zeolites* **1997**, *19*, 279. 10.1016/S0144-2449(97)00066-3.
6. Jia, W.; Chu, J.; Gong, J.; Yang, F.; Wang, S.; Xiong, S. Hydrothermal Synthetic Strategies of $Pt(NH_3)_2(NO_2)_2$ with Various Morphologies. *Mater. Lett.* **2024**, *358*, 135802. 10.1016/j.matlet.2023.135802.
7. Zhou, J.-H.; Sui, Z.-J.; Zhu, J.; Li, P.; Chen, D.; Dai, Y.-C.;Yuan, W.-K. Characterization of Surface Oxygen Complexes on Carbon Nanofibers by TPD, XPS and FT-IR. Carbon **2007**, *45*, 785. 10.1016/j.carbon.2006.11.019.
8. Hasegawa, G.; Deguchi, T.; Kanamori, K.; Kobayashi, Y.; Kageyama, H.; Abe, T.; Nakanishi, K. High-Level Doping of Nitrogen, Phosphorus, and Sulfur into Activated Carbon Monoliths and Their Electrochemical Capacitances. *Chem. Mater.* **2015**, *27*, 4703. 10.1021/acs.chemmater.5b01349.
9. van Beek, W.; Safonova, O.V.; Wiker, G.; Emerich, H. SNBL, a Dedicated Beamline for Combined In Situ X-ray Diffraction, X-ray Absorption and Raman Scattering Experiments. *Ph. Transit.* **2011**, *84*, 726. 10.1080/01411594.2010.549944.
10. Giulimondi, V.; Ruiz-Ferrando, A.; Giannakakis, G.; Surin, I.; Agrachev, M.; Jeschke, G.; Krumeich, F.; López, N.; Clark, A.H.; Pérez-Ramírez, J. Evidence of Bifunctionality of Carbons and Metal Atoms in Catalyzed Acetylene Hydrochlorination. *Nat. Commun.* **2023**, *14*, 5557. 10.1038/s41467-023-41344-0.
11. Newville, M. IFEFFIT: Interactive XAFS Analysis and FEFF Fitting. *J. Synchrotron. Radiat.* **2001**, *8*, 322. 10.1107/S0909049500016964.
12. Carberry, J.J. Physico-Chemical Aspects of Mass and Heat Transfer in Heterogeneous Catalysis. In Catalysis – Science and Technology. *Springer-Verlag*, **1987**, United States of America.

13. Mears, D. Diagnostic Criteria for Heat Transport Limitations in Fixed Bed Reactors. *J. Catal.* **1971**, *20*, 127. 10.1016/0021-9517(71)90073-X.
14. Weisz, P.B.; Prater, C.D. Interpretation of Measurements in Experimental Catalysis. *Adv. Catal.* **1954**, *6*, 143. 10.1016/S0360-0564(08)60390-9.
15. Kresse, G.; Furthmüller, J. Efficient Iterative Schemes for Ab Initio Total-Energy Calculations Using a Plane-Wave Basis Set. *Phys. Rev. B* **1996**, *54*, 11169. 10.1103/PhysRevB.54.11169.
16. Kresse, G.; Furthmüller, J. Efficiency of Ab Initio Total Energy Calculations for Metals and Semiconductors Using a Plane-Wave Basis Set. *Comput. Mater. Sci.* **1996**, *6*, 15. 10.1016/0927-0256(96)00008-0.
17. Perdew, J.P.; Burke, K.; Ernzerhof, M. Generalized Gradient Approximation Made Simple. *Phys. Rev. Lett.* **1996**, *77*, 3865. 10.1103/PhysRevLett.77.3865.
18. Grimme, S.; Antony, J.; Ehrlich, S.; Krieg, H. A Consistent and Accurate Ab Initio Parametrization of Density Functional Dispersion Correction (DFT-D) for the 94 Elements H-Pu. *J. Chem. Phys.* **2010**, *132*, 154104. 10.1063/1.3382344.
19. Kresse, G.; Joubert, D. From Ultrasoft Pseudopotentials to the Projector Augmented-Wave Method. *Phys. Rev. B* **1996**, *59*, 1758. 10.1103/PhysRevB.59.1758.
20. Blöchl, P.E. Projector Augmented-Wave Method. *Phys. Rev. B* **1994**, *50*, 17953. 10.1103/PhysRevB.50.17953.
21. Makov, G.; Payne, M.C. Periodic Boundary Conditions in Ab Initio Calculations. *Phys. Rev. B* **1995**, *51*, 4014. 10.1103/PhysRevB.51.4014.
22. Campbell, C.T.; Sellers, J.R. The Entropies of Adsorbed Molecules. *J. Am. Chem. Soc.* **2012**, *134*, 18109. 10.1021/ja3080117.
23. European Parliament & Council. Regulation (EC) No 1272/2008 of the European Parliament and of the Council of 16 December 2008 on Classification, Labelling and Packaging of Substances and Mixtures, Amending and Repealing Directives 67/548/EEC and 1999/45/EC, and Amending Regulation (EC) No 1907/2006. *OJEU* **2008**, 1. https://eur-lex.europa.eu/eli/reg/2008/1272/oj.
24. European Chemicals Agency (ECHA). https://echaeuropaeu/information-on-chemicals.
25. Examples of Common Laboratory Chemicals and Their Hazard Class. National Institutes of Health (NIH). https://orfodnihgov/EnvironmentalProtection/WasteDisposal/Pages/Examples+of+Common+Laboratory+ChemicalsandtheirHazardClassaspx.
26. Farr, J.; Gorman, D.; Sliva, D.; Hielscher, A.; Nguyen, T.; Baran, G.; Drake, B.; Ford, E.; Frurip, D.; Mulligan, K.; Ryan, J.W.; Viveros, D. Expanded Chemical Reactivity Worksheet (CRW4) for Determining Chemical Compatibility, Past, Present, and Future. *Process Saf. Prog.* **2017**, *36*, 24. 10.1002/prs.11833.
